# A New Role of Class A Hepatitis B Virus Capsid Assembly Modulators in Core Protein Dynamics and Covalently Closed Circular DNA Replenishment

**DOI:** 10.1016/j.jcmgh.2026.101860

**Published:** 2026-08-12

**Authors:** Chunkyu Ko, Xue Zhou, Romina Bester, Sehee Park, Jihyun Park, Christoph Blossey, Verena Plank, Samuel Hofmann, Michael Nassal, Sabrina Schreiner-Gruber, Lu Gao, Ulrike Protzer

**Affiliations:** 1 Institute of Virology, Technical University of Munich, School of Medicine and Health/Helmholtz Munich, Munich, Germany; 2 Infectious Diseases Therapeutic Research Center, Korea Research Institute of Chemical Technology (KRICT), Daejeon, Republic of Korea; 3 Roche Innovation Center Shanghai, Shanghai, China; 4 Department of Internal Medicine II, Molecular Biology, University Hospital Freiburg, Freiburg, Germany; 5 Institute of Virology, University Hospital Freiburg, Freiburg, Germany; 6 German Center for Infection Research (DZIF), Munich Partner Site, Munich, Germany

**Keywords:** Capsid, Capsid Assembly Modulators, Core Protein, Hepatitis B Virus

## Abstract

**Background & Aims:**

Current treatments of chronic hepatitis B are rarely curative. Capsid assembly modulators target capsid formation by the hepatitis B virus core protein. Class A capsid assembly modulators induce abnormal core protein assembly, but how they affect core protein dynamics and inhibit hepatitis B virus replication is only partially understood.

**Methods:**

Human liver chimeric mice and hepatitis B virus–susceptible cells were infected to monitor dynamic changes and the fate of core protein and assembled capsids. We analyzed hepatitis B virus and protein dynamics over 4 weeks under treatment of the class A capsid assembly modulator molecule HAP_R01.

**Results:**

We found an altered nuclear-cytoplasmic distribution of hepatitis B virus core protein upon treatment with HAP_R01. This effect was confirmed in primary human hepatocytes and in hepatitis B virus–infected liver-humanized mice. Mechanism-of-action studies in hepatitis B virus–permissive cells demonstrated that HAP_R01 primarily targets newly synthesized core protein and affects capsid assembly, core protein localization, and solubility in a dose- and time-dependent manner. At 50 nM, HAP_R01 promoted assembly of hepatitis B virus genome-free capsids and nuclear accumulation of core protein. At ≥500 nM, HAP_R01 treatment reduced soluble core protein and capsid levels, but markedly increased insoluble core protein levels, resulting in an overall intracellular accumulation of core protein. Insoluble nuclear agglomerations of core protein were deposited in promyelocytic leukemia nuclear bodies. Treating infected cells for 31 days significantly reduced an established covalently closed circular DNA pool and secreted hepatitis B surface antigen and hepatitis B e antigen levels, by inhibiting covalently closed circular DNA replenishment.

**Conclusions:**

As an exemplary class A capsid assembly modulator, HAP_R01 inhibits hepatitis B virus genome replication by perturbing capsid assembly and by inducing insoluble core protein accumulation in the nucleus that affects replenishment of the covalently closed circular DNA pool.

## Graphical abstract

**Figure:**
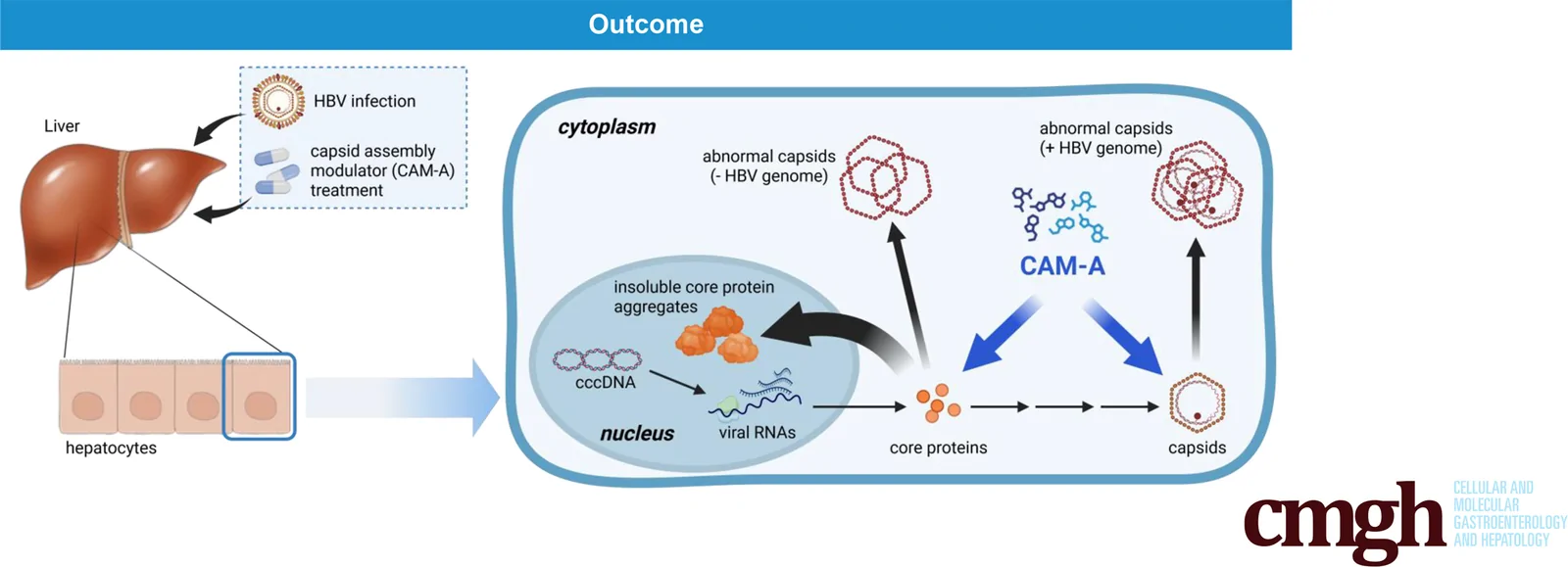

**Summary**

This study describes a new antiviral mechanism whereby class A capsid assembly modulators redirect hepatitis B virus core protein to the nucleus, forming insoluble aggregates that eventually trigger the loss of established hepatitis B virus covalently closed circular DNA.

**What You Need to Know**

**Background**

Capsid assembly modulators act on the hepatitis B virus core protein to block normal nucleocapsid formation. However, their effects on core protein fate and viral persistence are not well-understood.

**Impact**

The class A capsid assembly modulator HAP_R01 drives newly synthesized hepatitis B virus core protein to the nucleus, where it forms insoluble aggregates in promyelocytic leukemia nuclear bodies, leading to disruption of covalently closed circular DNA maintenance.

**Future Directions**

By misdirecting capsid assembly to the nucleus, capsid assembly modulators can control established hepatitis B virus infection, highlighting the core protein as a promising therapeutic target for chronic hepatitis B.

Hepatitis B virus (HBV) is an enveloped hepatotropic virus with a partially double-stranded relaxed circular (rc)DNA within its icosahedral capsid assembled from core protein dimers.^1^ Although most people infected in adulthood have an acute and self-limiting HBV infection, HBV causes chronic infection in up to 90% of infected infants.^2^ An estimated 254 million people worldwide are chronically infected with HBV, and these chronic carriers are at high risk for developing liver diseases, such as liver cirrhosis and hepatocellular carcinoma.^3^ Viral hepatitis has become the seventh leading cause of death worldwide, with 1.1 million annual HBV-related deaths.^3^^,^^4^

Following infection, the rcDNA is imported to the nucleus and converted to covalently closed circular DNA (cccDNA). cccDNA serves as the transcriptional template for all viral RNAs, including pregenomic RNA (pgRNA), the template for viral reverse transcription, and thus represents the viral persistence reservoir.^5^ Once established, intrahepatic cccDNA pools are continuously replenished by new rounds of infection and intracellular retargeting of HBV capsid containing the viral genome to the nucleus.^6^ Nucleos(t)ide analogs (NUCs) as the currently available antivirals inhibiting the reverse transcriptase (RT) of HBV cannot eliminate cccDNA. Although current NUC therapy is well-tolerated and effectively suppresses viral DNA synthesis, the error-prone nature of HBV polymerase can lead to the emergence of drug-resistant variants over time.^7^ Thus, new therapeutic strategies against alternative targets, which are curative on their own or complement existing regimens, are urgently needed.

Therapeutic strategies for HBV infection can be divided into directly acting antivirals and immunomodulators.^8^ Immune modulation aims at restoring HBV-specific cellular and humoral immunity that would eventually control HBV. Direct antiviral strategies aim at targeting the virus itself, based on a better understanding of the HBV life cycle and virus–host interaction. Given that HBV core protein and capsids are involved in almost every step of the virus life cycle, they are attractive viral targets, providing a new antiviral platform, the capsid assembly modulators (CAMs).^9^ Distinct chemical classes of HBV core protein–targeting small molecules have been identified and are under preclinical and early clinical development.^8^^,^^10^ Inhibiting capsid function can also help controlling human immunodeficiency virus (HIV) infection, and the first-in-class HIV capsid inhibitor lenacapavir was recently approved by the United States Food and Drug Administration for treatment of multidrug-resistant HIV-1.^11^

CAMs can be classified into 2 classes based on their phenotypic effects on capsid formation in vitro: CAM-E (empty) and CAM-A (aberrant).^12^ CAM-E compounds such as phenylpropenamides (AT-130 and AT-61 as examples) and sulfamoylbenzamides (SBAs; DVR-23 and SBA_R01 as examples) interfere with pgRNA encapsidation, although they still allow formation of morphologically normal or near-normal capsids.^13^, ^14^, ^15^ Heteroaryldihydropyrimidines (HAPs; Bay41-4109 and GLS4 as examples) belong to the CAM-A subclass, promote the assembly of noncapsid core protein polymers in vitro, and induce the depletion of core proteins in cell culture and in vivo.^16^, ^17^, ^18^, ^19^ Recent studies have shown that CAM-A compounds induce nuclear accumulation of core protein aggregates, which can result in apoptotic hepatocyte death both in vivo and in cell culture.^20^^,^^21^ However, this apoptotic elimination of hepatocytes primarily occurs when cells express high levels of core protein.^22^^,^^23^ This raised the question of whether the consequence of CAM-A treatment is influenced by experimental conditions highlighting the importance of closely monitoring core protein dynamics and fate, particularly under de novo infection conditions.

HAP_R01 is a potent HAP-type CAM-A that binds to core protein dimer–dimer interface and disrupts proper capsid assembly with double-digit nanomolar half-maximal effective concentration (EC_50_) values against HBV replication.^15^^,^^24^

We previously reported that HAP_R01 destabilizes incoming capsids, inhibits cccDNA formation early in HBV infection,^24^ and suppresses biosynthesis of the secretory hepatitis B e antigen (HBeAg),^25^ which shares most of its amino acid sequence with core protein. In this study, we demonstrated that core protein and capsids follow dynamic changes during the course of HAP_R01 treatment both in vivo and in vitro models allowing for a de novo HBV infection. HAP_R01 promoted a redistribution of newly synthesized core protein to the nucleus where it accumulated in promyelocytic leukemia nuclear bodies (PML-NBs) and became insoluble. This effect was more pronounced at concentrations of ≥500 nM. At a lower concentration (50 nM in cell culture), HAP_R01 also led to intracellular accumulation of HBV genome-free capsids. Accordingly, total intracellular core protein levels were even modestly increased upon HAP_R01 treatment, indicating that core protein degradation and cell death plays a minimal role. Furthermore, we showed that continuous HAP_R01 treatment over 4 weeks was able to reduce an established cccDNA pool and serum hepatitis B surface antigen (HBsAg) levels.

## Results

### HAP_R01 has Broad Antiviral Activity Against Nucleos(t)ide-Resistant Mutants and Different Hepatitis B Virus Genotypes

We were interested to know whether HAP_R01 could suppress viral genome replication driven by NUC-resistant HBV mutants (Figure 1*A*). Two mutants in the RT domain of HBV polymerase showing resistance to lamivudine (LMV) and entecavir (ETV) were chosen for this study: (1) rtL180M/rtM204V and (2) rtI169T/rtL180M/rtM204V/rtM250V (Figure 1*B*). We transfected a replication-competent HBV plasmid containing either wild-type (WT) or a NUC-resistant HBV genome into HepG2 cells, treated with increasing concentrations of HAP_R01 or NUCs, and measured capsid-associated HBV-DNA by Southern blot analysis (Figure 1*C*). For WT HBV, ETV markedly reduced the amounts of rcDNA and replication intermediates (EC_50_, 4 nM), whereas LMV showed a 2-log lower antiviral activity (EC_50_, 445 nM). HAP_R01 inhibited viral DNA synthesis with an EC_50_ value of 77 nM. As expected, rtL180M/rtM204V and rtI169T/rtL180M/rtM204V/rtM250V substitutions reduced sensitivity to ETV by 10-fold (EC_50_, 40 nM) and more than 100-fold (EC_50_, 481 nM), respectively, and showed complete resistance to LMV. In contrast, treating cells with HAP_R01 diminished viral DNAs at comparable levels to the WT HBV (EC_50_, 154 nM to rtL180M/rtM204V and EC_50_, 85 nM to rtI169T/rtL180M/rtM204V/rtM250V).

**Figure 1:**
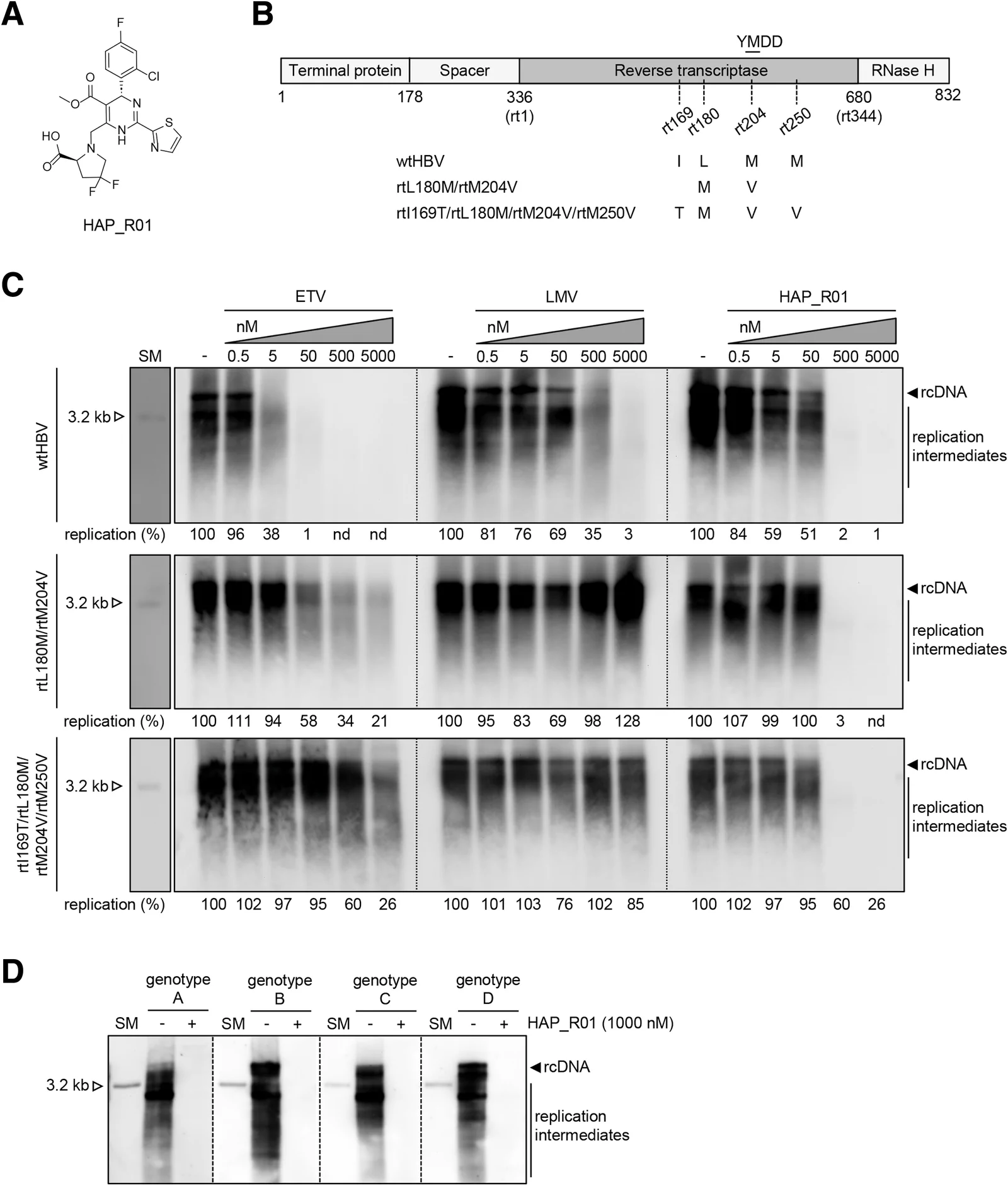
**Effect of HAP_R01 on antiviral drug-resistant HBV mutants and different HBV genotypes.** (*A*) Chemical structure of HAP_R01. (*B*) Domain organization of wild-type (wt) HBV polymerase (genotype D, subtype ayw) and drug-resistant mutants. The 4 subdomains terminal protein, spacer, RT with the YMDD active site motif, and RNase H, and their approximate borders are indicated by amino acid numbers. Mutations conferring drug-resistance amino acid are indicated by RT domain-specific numbering. (*C*) HepG2 cells were transfected with HBV replication-competent plasmid (pCH9_3091) harboring either wt polymerase or the rt mutants. One day post-transfection cells were treated with increasing concentrations (0–5000 nM) of antivirals for 3 days as indicated. HBV-DNA (rcDNA and replication intermediates) was extracted from intracellular capsids and subjected to Southern blotting using an HBV-DNA probe. A 3.2-kb full-length linear HBV-DNA served as a size marker. Densitometric quantification of all forms of viral DNAs is shown below the blots. (*D*) HepG2 cells were transfected with HBV expression plasmids containing different HBV genotypes as indicated. At 1 day post-transfection, cells were either mock-treated (DMSO only) or treated with HAP_R01 (1 μM) for 3 days. Intracellular capsid-associated DNA was detected by Southern blot analysis. SM, size marker.

Next, we wondered if HAP_R01 was able to inhibit viral genome replication of the major HBV genotypes circulating worldwide (genotype A, B, C and D). As shown in Figure 1*D*, HAP_R01 was equally effective in suppressing viral DNA synthesis by all 4 genotypes. Overall, this demonstrated broad antiviral activity of HAP_R01 against NUC-resistant mutants and different HBV genotypes, encouraging us to evaluate the antiviral activity and potency of HAP_R01 in vivo.

### HAP_R01 Inhibits Hepatitis B Virus Viremia and Alters Subcellular Localization and Expression of Core Protein In Vivo

We employed human liver chimeric urokinase-type plasminogen activator/severe combined immunodeficiency (uPA/SCID) mice permissive for HBV infection to evaluate HAP_R01 antiviral activity in vivo. Eight weeks after HBV infection, mice were randomized into 4 groups receiving 2 weeks oral administration of HAP_R01 (3 mg/kg twice daily), HAP_R01 (10 mg/kg twice daily), tenofovir disoproxil fumarate (TDF) (5 mg/kg once daily), or vehicle control, respectively. Two weeks treatment with HAP_R01 at 3 and 10 mg/kg twice daily reduced serum HBV-DNA levels by 1.3- and 2.7-log, respectively (Figure 2*A*). In the mice administered with TDF (5 mg/kg once daily), a 2.3-log reduction in viral load was observed. Due to the short duration of treatment, neither intrahepatic HBV-DNA, including cccDNA (Figure 2*B*), nor serum HBsAg and HBeAg levels showed significant differences by either HAP_R01 or TDF treatment (Figure 2*C* and *D*). Immunohistochemical staining of liver sections for HBV core protein showed that, in TDF-treated mice, core protein was mainly located in the cytoplasm, if at all, and a diffuse nuclear staining was observed (Figure 2*E*). In contrast, in HAP_R01-treated mice, HBV core protein showed a predominant nuclear distribution, especially in the high dose (10 mg/kg twice daily) group, where we also noted a reduction of cytoplasmic core protein staining. Taken together, our in vivo observations highlighted the antiviral activity of HAP_R01 on HBV replication and indicated that high-dose treatment affects the subcellular localization and levels of core protein, prompting us to investigate the underlying molecular mechanisms.

**Figure 2:**
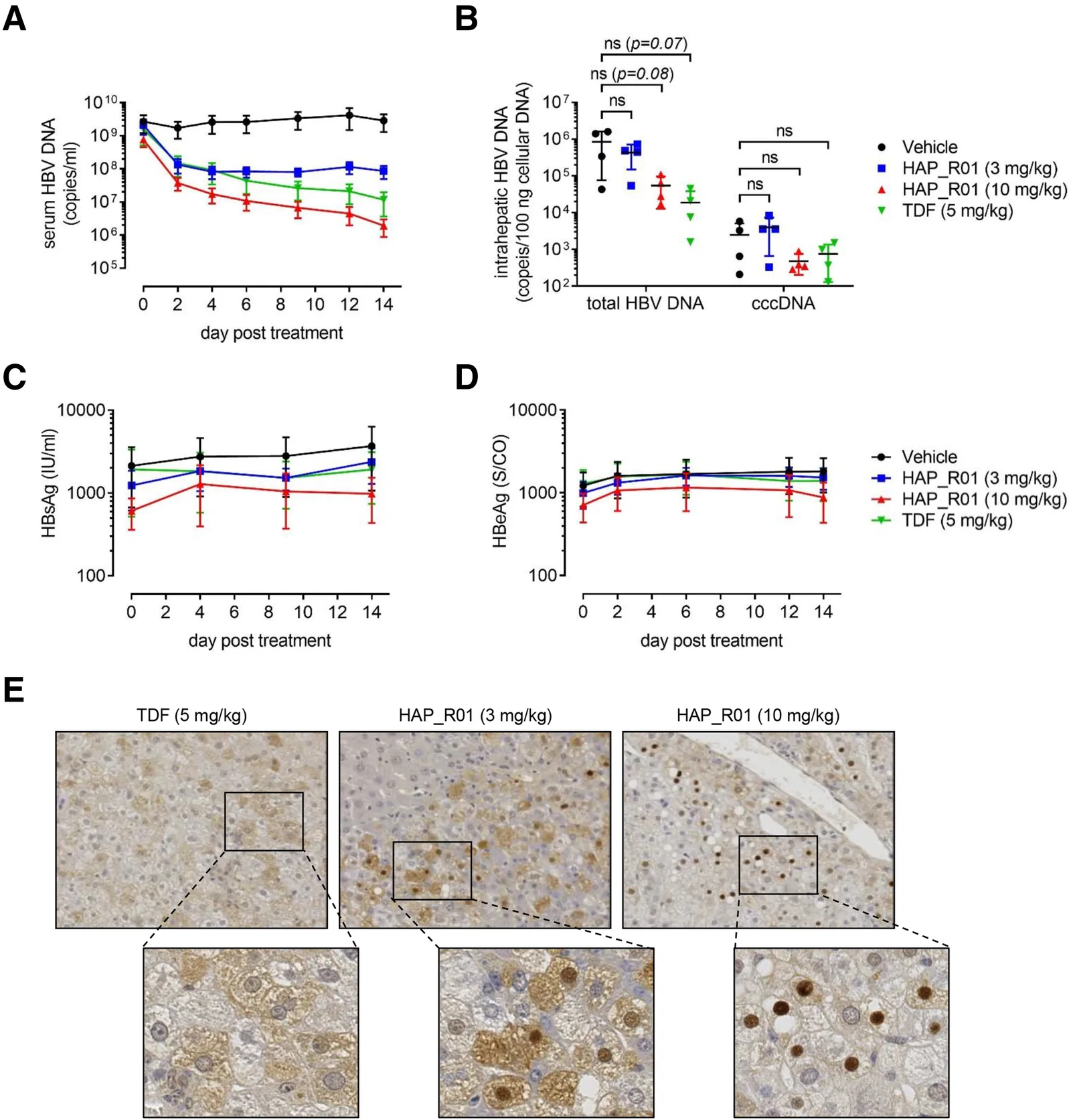
**In vivo activity of HAP_R01 in human liver chimeric mice HBV-infected (genotype C) human liver chimeric uPA/SCID mice (4 mice per group) were treated with HAP_R01 (3 or 10 mg/kg, twice daily), vehicle control (twice daily), or TDF (5 mg/kg, once daily) for 2 weeks.** (*A*) Serum HBV-DNA and (*B*) total intrahepatic HBV-DNA and cccDNA levels were quantified by qPCR. Values are shown as mean ± standard error of the mean (n = 4). (*C*) Serum HBsAg and (*D*) HBeAg levels were measured by using chemiluminescence enzyme Immunoassays. Data are presented as mean ± standard error of the mean (n = 4). (*E*) Intrahepatic HBV core protein was visualized by immunohistochemical staining. Magnification, ×200. Enlarged views of the marked regions in each image are shown. Similar staining patterns were observed in other mice in each group. ns, not significant; significance indicated by *P* value derived from the Student *t* test.

### HAP_R01 Inhibits Hepatitis B Virus Replication and Exhibits a Dual Effect on Capsid Assembly and Localization of Hepatitis B Virus Core Protein

To examine the antiviral mechanisms of HAP_R01, we employed cell-culture models allowing for infection with HBV. Considering that fully establishing HBV cccDNA takes 3 days^6^ and that HAP_R01 inhibits de novo cccDNA formation when applied during and early after HBV inoculation,^24^ we started treatment of HepG2-NTCP-K7 cells with increasing concentrations of HAP_R01 only at day 3 after cccDNA formation and analyzed viral parameters at day 9. Secretion of progeny virus HBV-DNA was reduced with an EC_50_ of 20 nM (Figure 3*A*). Southern blot analysis of capsid-associated HBV-DNA showed a similar dose-related reduction (Figure 3*B, upper panel*) with an EC_50_ value of 40 nM, indicating the inhibitory role of HAP_R01 on viral DNA replication. Surprisingly, we found that, depending on its concentration, HAP_R01 had distinct effects on HBV replicative intermediates (Figure 3*B, upper panel*) and cytoplasmic HBV core protein, without affecting viral envelope protein (Figure 3*B, lower panels*), HBV RNA (Figure 3*C*), or HBV cccDNA levels (Figure 3*D*). This indicated a selected, core protein–targeted antiviral activity.

**Figure 3:**
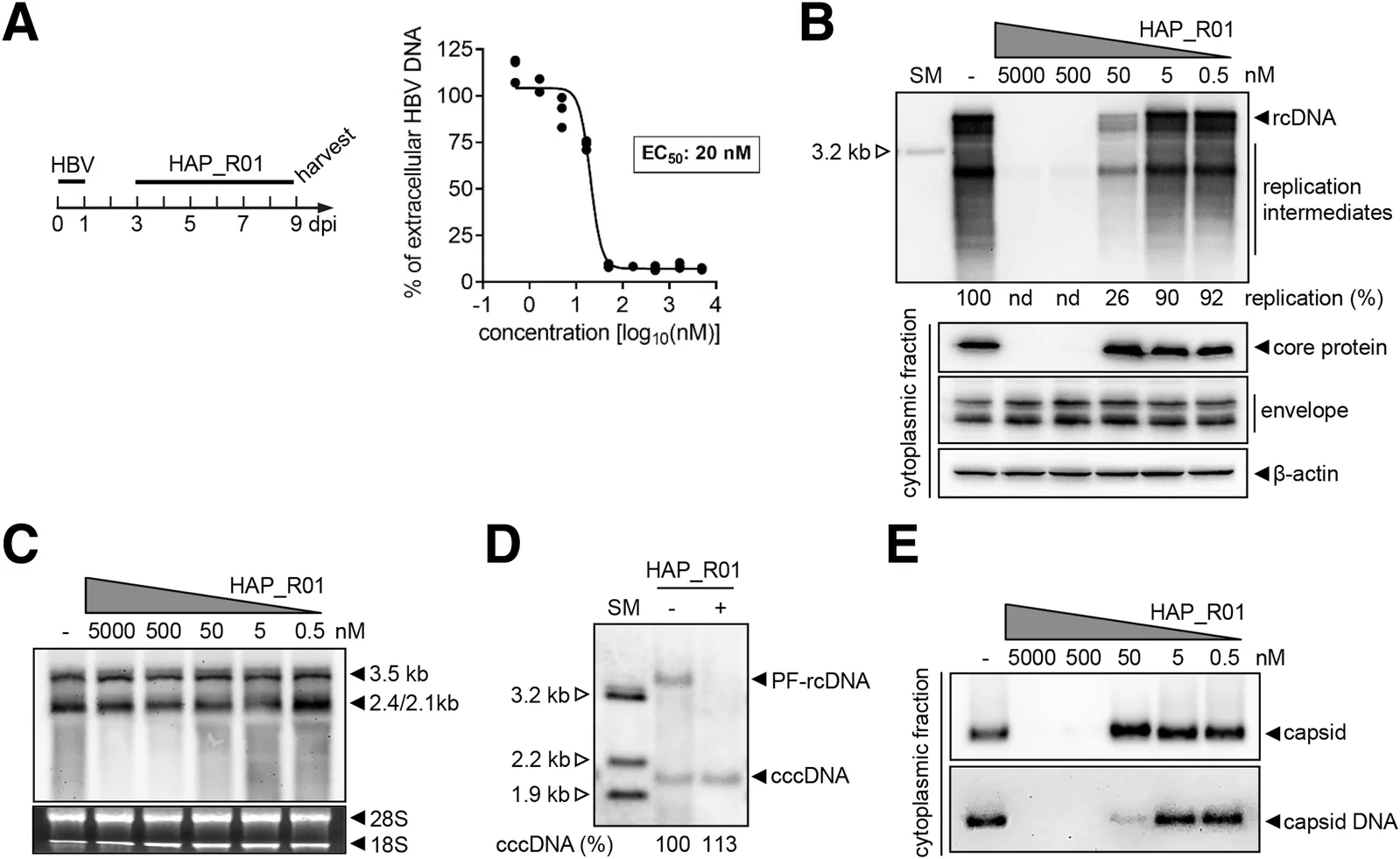
**Effects of HAP_R01 on HBV-DNA replication, capsid assembly, and core protein localization in *de novo* HBV-infected cells.** HepG2-NTCP-K7 cells were infected with HBV at a multiplicity of infection (moi) of 1000 vp/cells and treated with various concentrations of HAP_R01 as indicated from 3 to 9 days PI. (*A*) HBV-DNA isolated from cell-culture media collected from 7 to 9 days PI was measured by qPCR in 3 parallel infections. The EC_50_ of HAP_R01 was determined by nonlinear regression analysis. (*B*) Cytoplasmic fractions were obtained and capsid-associated HBV-DNA was detected by Southern blot analysis using an HBV-DNA probe and a linear 3.2-kb HBV-DNA fragment as size marker. Core and envelope protein expression was detected by Western blot analysis. β-actin served as a loading control. (*C*) Total intracellular HBV-RNA was subjected to Northern blot analysis using an HBV-DNA probe. Ribosomal RNAs (28S and 18S) are shown as loading controls. (*D*) Protein-free DNA was extracted using a modified Hirt method and subjected to Southern blot analysis. Protein-free rcDNA (PF-rcDNA) and cccDNA are indicated. Double-stranded linear HBV-DNA fragments (3.2, 2.2 and 1.9 kb) were used as size markers. (*E*) Cytoplasmic HBV capsids were analyzed by native agarose gel electrophoresis followed by immunoblotting with an anti-core serum and in situ hybridization using an HBV-DNA probe. SM, size marker; vp, enveloped DNA containing virus particles.

Native agarose gel electrophoresis analysis of HBV capsids isolated from the cytoplasm of HAP_R01-treated cells (Figure 3*E*) revealed that, at 50 nM HAP_R01, viral DNA in HBV capsids was already markedly reduced, although capsid band intensity was modestly increased, and capsids showed a slower migration pattern than capsids from nontreated cells. At higher HAP_R01 concentrations, no cytoplasmic capsids were detected. Similar HAP_R01 concentration-dependent effects were seen in HepAD38 and HepG2.2.15 cells (Figure 4*A*) in which viral replication is derived from integrated HBV genomes and were confirmed by analyzing capsid and HBV-DNA contents after cesium chloride density gradient fractionation (Figure 4*B*).

**Figure 4:**
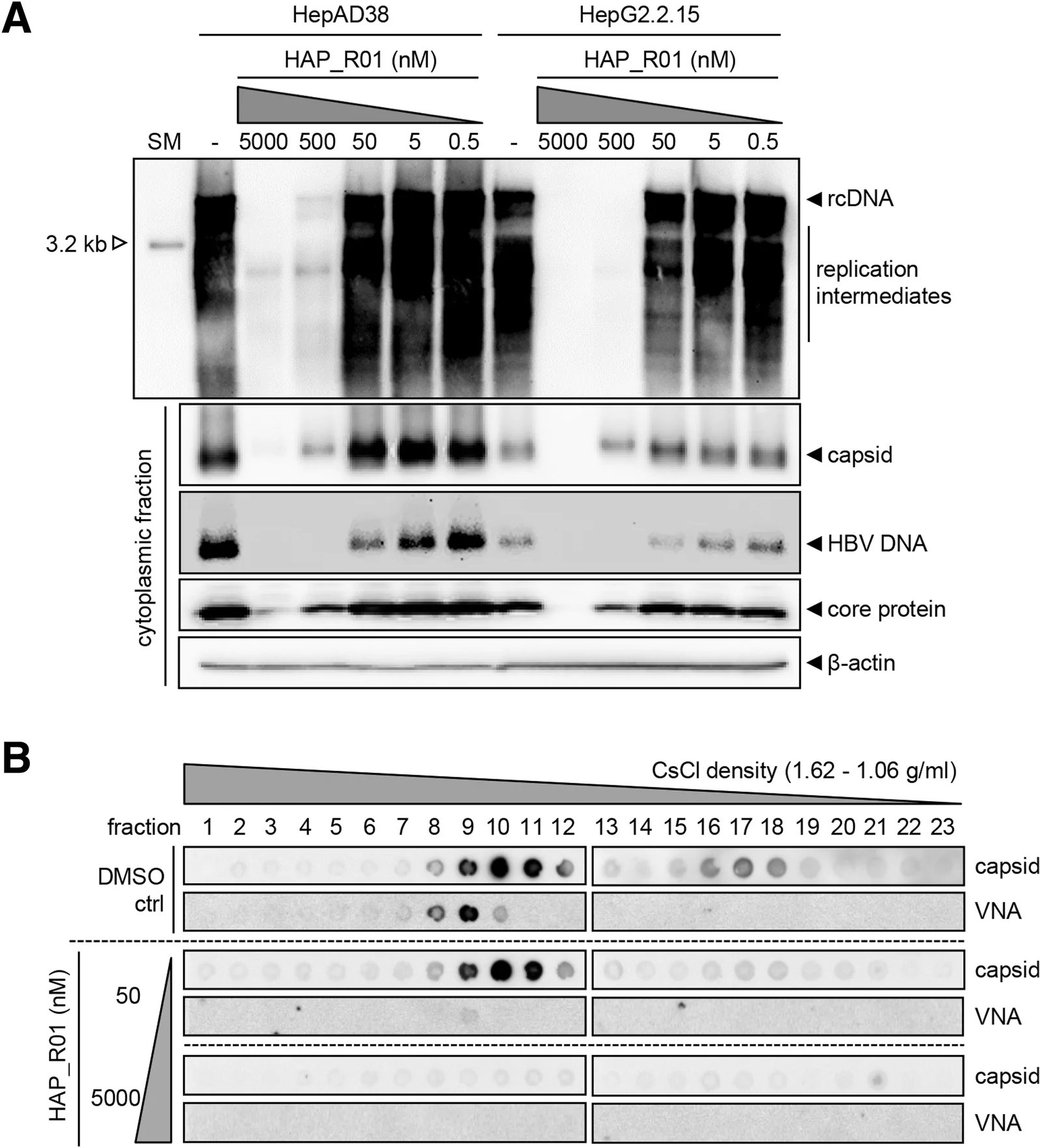
**Effect of HAP_R01 on viral DNA replication, capsid assembly, and core protein expression in HBV-replicating cell lines.** (*A*) HepAD38 and HepG2.2.15 cells were treated with decreasing concentrations of HAP_R01 for 5 days. Intracellular capsid-associated HBV-DNA (rcDNA and replication intermediates) were analyzed by Southern blotting with an HBV-DNA probe. A 3.2-kb HBV-DNA fragment serves as a size marker. Capsid was analyzed by native agarose gel electrophoresis using an anti-core serum. HBV-DNA within capsid was subsequently detected by in situ hybridization using an HBV-DNA probe. Core protein was detected by Western blot analysis. β-actin serves as a loading control. (*B*) HepG2-NTCP-K7-H1.3Lˉ cells were either mock-treated (DMSO) or treated with HAP_R01 (50 or 5000 nM) for 3 days. Cytoplasmic cell lysates were subjected to cesium chloride density gradient centrifugation. Twenty-three fractions (from bottom to top; 1.5 mL per fraction) were collected, and 10 μl of each fraction was transferred onto a polyvinylidene fluoride membrane by dot-blot procedure. Levels of capsid were analyzed by immunoblotting. Viral nucleic acids were subsequently detected by incubating the same membrane with an HBV-DNA probe. Fractions 8 to 10 contain HBV-DNA-containing capsids, whereas fractions 11 and 12 contain only empty capsids. CsCl, cesium chloride; SM, size marker; VNA, viral nucleic acids.

The effect of HAP_R01 following treatment cessation was investigated by treating infected cells only for 3 days, followed by analysis of viral parameters every 3 days up to 15 days postwithdrawal (Figure 5*A*). Intracellular HBV-DNA was undetectable until day 12 and was only marginally detectable at day 15, indicating a prolonged antiviral effect (Figure 5*B, upper panel*). Cytoplasmic core protein and HBV genome-free capsids reappeared at day 6 and gradually recovered but did not reach levels observed in untreated cells (Figure 5*B, middle panels*), whereas HBV RNA remained unchanged (Figure 5*B, lower panels*). Notably, this effect was more pronounced than that observed with other CAMs (Bay41-4109 and AT130) and NUCs (ETV), where HBV-DNA containing capsids were either completely or partially restored to levels comparable to untreated cells (Figure 5*C*).

**Figure 5:**
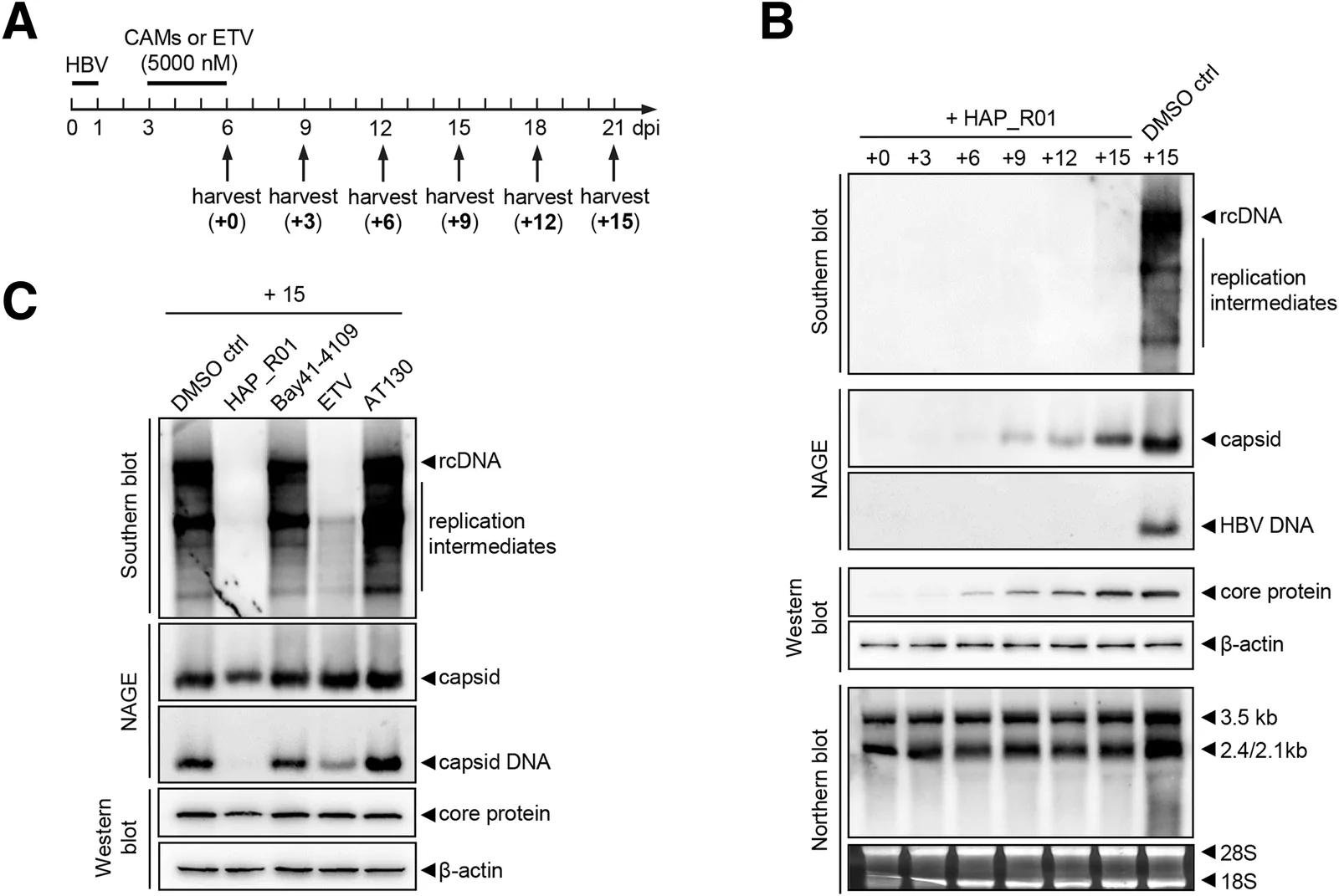
**Antiviral effects of HAP_R01 after treatment withdrawal.** (*A*) Experimental set-up. HBV-infected HepG2-NTCP-K7 cells were either DMSO-treated or treated with HAP_R01 (5 μM) for 3 days from day 3 PI. After extensive washing, cells were maintained in drug-free media and harvested at indicated timepoints. (*B*) Intracellular capsid-associated DNA, including rcDNA and replication intermediates, was isolated and detected by Southern blot hybridization using an HBV-DNA probe. Capsid and associated DNA were examined by native agarose gel electrophoresis (NAGE). Core protein expression was detected by Western blotting. Viral RNA species were determined by Northern blot analysis using an HBV-DNA probe. β-actin and ribosomal RNAs (28S and 18S) served as loading controls. (*C*) HBV-infected HepG2-NTCP-K7 cells were treated with the indicated antivirals (5 μM, each) and maintained in the absence of antivirals as in panel (*A*). At day 15 post-drug withdrawal, viral parameters were analyzed as described in panel (*B*).

Taken together, our findings indicated that HAP_R01 maintains antiviral activity after treatment withdrawal and that HAP_R01 promotes structurally altered HBV genome–free capsid formation (ie, empty capsid) by either hampering pgRNA packaging or subsequent reverse transcription and—at higher concentrations—the intracellular loss or subcellular redistribution of HBV capsids.

Therefore, we looked at core protein expression and subcellular localization in HBV-infected HepG2-NTCP-K7 cells by immunofluorescence staining. In cells treated with dimethyl sulfoxide (DMSO) alone or a low-dose (5 nM) HAP_R01, the core protein signals were predominantly located in the cytoplasm (Figure 6*A*). At higher doses, HAP_R01 caused a redistribution of core protein to the nucleus, with formation of core protein—specific foci. At high concentrations (500 and 5000 nM), the cytoplasmic core protein signals decreased, leaving bigger and more intense core protein foci exclusively in the nucleus. HAP_R01 treatment of de novo HBV-infected primary human hepatocytes and Huh7-NTCP cells confirmed this finding (Figure 6*B*). Continuous treatment with HAP_R01 for up to 27 days resulted in the formation of larger nuclear core protein foci in a reduced number of infected cells over time (Figure 6*C*).

**Figure 6:**
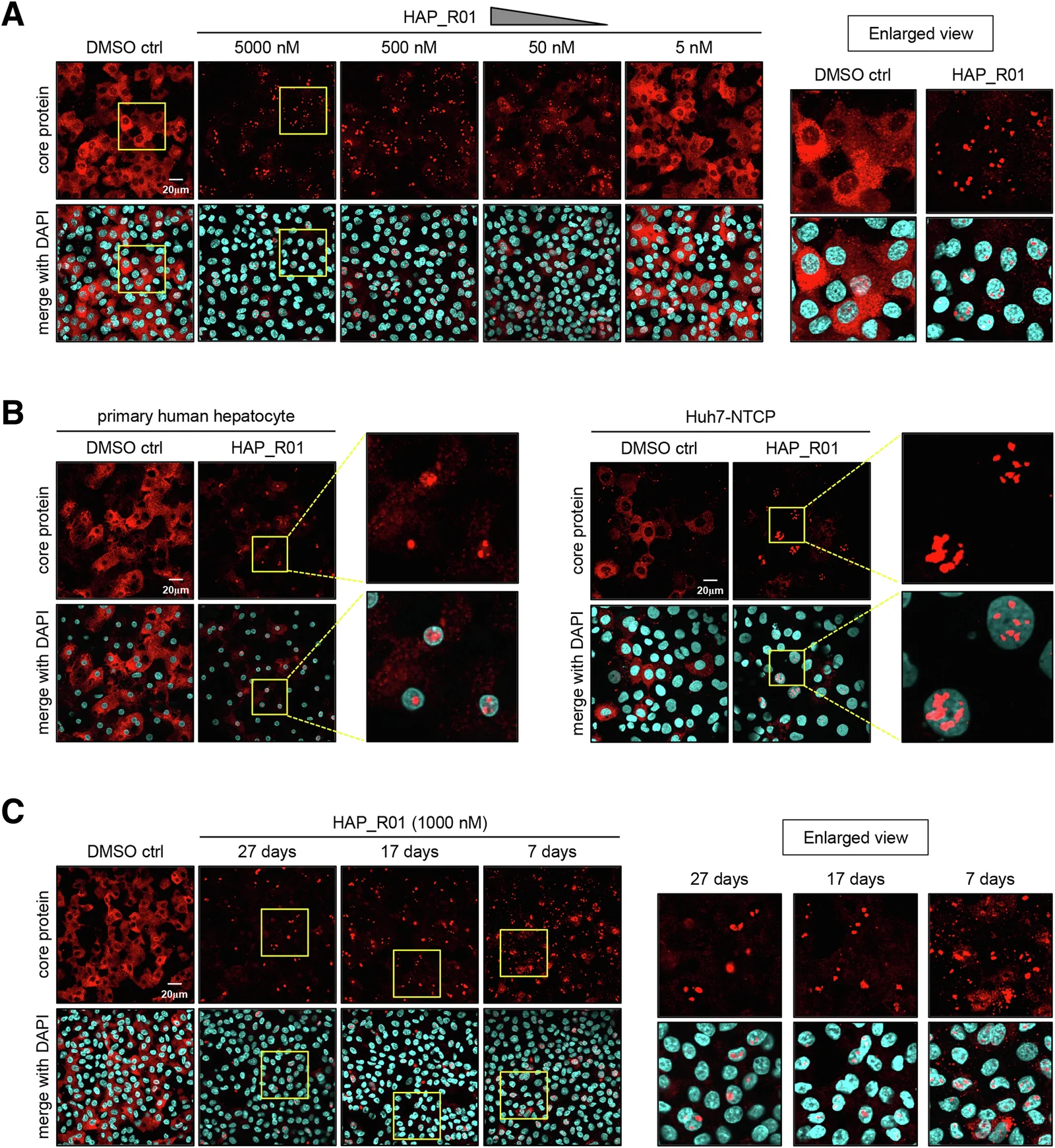
**Effects of HAP_R01 on HBV core protein expression and subcellular localization.** Cells were infected with or without HBV at a multiplicity of infection (moi) of 1000 vp/cells. (*A*) HBV-infected HepG2-NTCP-K7 cells were treated with decreasing concentrations of HAP_R01 for 6 days starting at 3 days PI. Cells were fixed and stained for HBV core protein (*red*) with an anti-core antibody. Nuclei were counterstained with 4′,6-diamidino-2-phenylindole (DAPI; *cyan*). *Enlarged views* of the boxed regions in DMSO- and 5000 nM HAP_R01-treated cells are shown. (*B*) Primary human hepatocytes and Huh7-NTCP cells were treated with 5000 nM HAP_R01 and stained for HBV core protein and nuclei. *Enlarged views* of the boxed regions highlight cells containing nuclear core protein foci. (*C*) HBV-infected cells were either mock-treated for 27 days or treated with HAP_R01 (1000 nM) for 7, 17, and 27 days starting at 3 days PI. Cells were stained for HBV core protein as described in panel (*A*). Scale bars, 20 μm. vp, enveloped DNA containing virus particles.

In summary, depending on the concentration and duration of treatment, HAP_R01 inhibits HBV replication by misdirecting capsid assembly, by reducing cytoplasmic core protein levels, and by inducing translocation of core protein to distinct nuclear structures.

### HAP_R01 Induces Dynamic Changes in Capsid and Core Protein Localization and Solubility

To understand how HAP_R01 affects capsid assembly and alters core protein localization, we treated HBV-infected cells with HAP_R01 at 50 or 5000 nM for 1 to 5 days and monitored the levels of soluble core protein and capsid levels in cytoplasm, nucleus, and insoluble protein (Figure 7*A*). At 50 nM, the amounts of cytoplasmic empty capsid increased over time (Figure 7*A, left*). We noted a slight upward mobility shift of the main capsid band, which was not observed for the capsid-associated HBV-DNA bands. Given that electrophoretic mobility changes reflect alterations in capsid structure,^26^ it is likely that HAP_R01 facilitated the production of structurally altered empty capsids but had a smaller or no impact on HBV-DNA–containing capsids that had already been assembled before HAP_R01 treatment. A modest elevation of capsid bands may reflect accelerated assembly and/or enhanced stability of newly forming capsids in the presence of HAP_R01.

**Figure 7:**
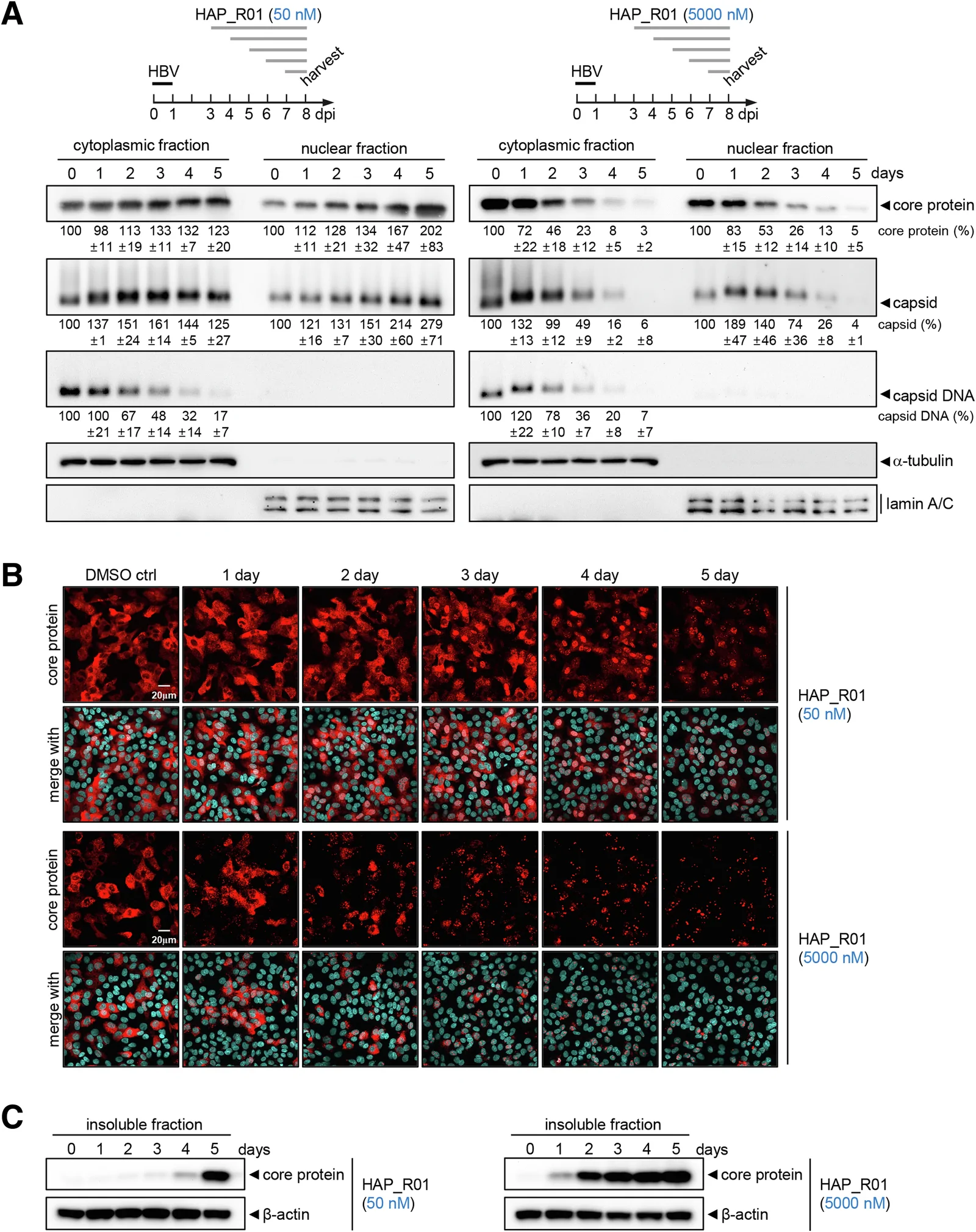
**Kinetic analysis of core protein and capsid localization, dynamics, and expression under HAP_R01 treatment.** HepG2-NTCP-K7 cells were infected with HBV at a multiplicity of infection (moi) of 300 vp/cells and treated with either 50 nM or 5000 nM HAP_R01 for the indicated times. Day 0 refers to the time of adding the HBV inoculum. (*A* and *C*) Cells were fractionated into soluble (cytoplasmic and nuclear) and insoluble fractions as described in Materials and Methods. Core protein was detected by Western blot analysis. Capsid and capsid-associated HBV-DNA were analyzed by native agarose gel electrophoresis using anti-core antiserum and an HBV-DNA probe, respectively. Representative data of 3 independent experiments are shown. Each band intensity was quantified and shown as mean ± standard deviation (n = 3). Tubulin and lamin A/C served as cytoplasmic and nuclear markers, respectively. (*B*) Cells were stained for HBV core protein using anti-core antiserum. Nuclei were stained with 4′,6-diamidino-2-phenylindole (DAPI). Scale bars, 20 μm. vp, enveloped DNA containing virus particles.

Interestingly, the levels of nuclear core protein and capsid gradually increased (Figure 7*A, right*), a phenomenon not reported previously. As described before,^27^ the nuclear capsids were empty and did not contain viral DNA. Immunofluorescence staining confirmed core protein accumulation in the nucleus and localization in distinct foci after 4 days of treatment (Figure 7*B, top*). Because core protein has both nuclear localization signals and nuclear export signals,^28^^,^^29^ this indicated an altered cytoplasmic–nuclear shuttling of core.

To examine the nature of the nuclear core protein foci, we solubilized proteins remaining in the pellet after harvesting soluble cytoplasmic and nuclear fractions and analyzed the levels of core protein in this insoluble fraction (Figure 7*C, left*). Western blotting detected insoluble core proteins that rapidly increased between day 4 and 5 of 50 nM HAP_R01 treatment, correlating with the amount of nuclear core protein foci (Figure 7*B, top*). Collectively, these results demonstrated that HAP_R01 misdirects core protein assembly into empty capsids and leads to core protein accumulation in the nucleus, possibly as insoluble aggregates accumulating in specific nuclear compartment(s).

At 5000 nM, HAP_R01 treatment rapidly reduced soluble cytoplasmic and nuclear core protein levels (Figure 7*A, right*) but increased its levels in the insoluble fraction (Figure 7*C, right*). The mobility shift and enhanced signal of both capsid and capsid-associated HBV-DNA bands at day 1 is most likely due to the targeting of preformed, rcDNA-containing HBV capsids by HAP_R01 that has been reported previously.^24^ The reduction of soluble core protein seemed to occur faster than the loss of deformed mature capsids, indicating that high-dose HAP_R01 targets newly synthesized core protein for insolubilization, secretion, or degradation. Visualizing HBV core protein by immunofluorescence confirmed a gradual decrease of cytoplasmic core protein signal with simultaneous formation of nuclear core protein foci already after 1 to 2 days of treatment (Figure 7*B, bottom*). In summary, our spatiotemporal analysis highlighted time- and concentration-dependent effects of HAP_R01, which result in dynamic changes of core protein and capsid levels, localization, and solubility.

### HAP_R01 Induces Core Protein Condensation in Promyelocytic Leukemia Nuclear Bodies Independent of Promyelocytic Leukemia Protein

Given that HAP_R01 triggered core protein condensation and aggregation without affecting viral RNA levels, we investigated whether aggregated core protein was secreted into the extracellular space. The analysis of denatured cell culture medium (Figure 8*A*) showed that HAP_R01 reduced the secretion of core protein according to what we had observed for progeny virus (Figure 1*A*). Thus, we investigated the fate of core protein upon HAP_R01 treatment and analyzed core protein levels in RIPA-soluble and -insoluble fractions and total cell lysate. High concentrations (≥500 nM) of HAP_R01 reduced soluble core protein levels, whereas insoluble core protein levels were markedly increased (Figure 8*B*). Notably, HAP_R01 even increased total intracellular core protein levels (Figure 8*C*). Similar results were obtained when treating infected cells with Bay41-4109 or GLS4 (Figure 8*D*), indicating condensation of core protein in insoluble fractions is a common mode-of-action shared by CAM-A compounds. Interestingly, although total intracellular monomeric core protein remained elevated by approximately 2- to 3-fold throughout the treatment period, additional high–molecular-weight and low–molecular-weight core protein species emerged after 12 days of treatment and progressively accumulated with continued exposure up to 24 days (Figure 8*E*).

**Figure 8:**
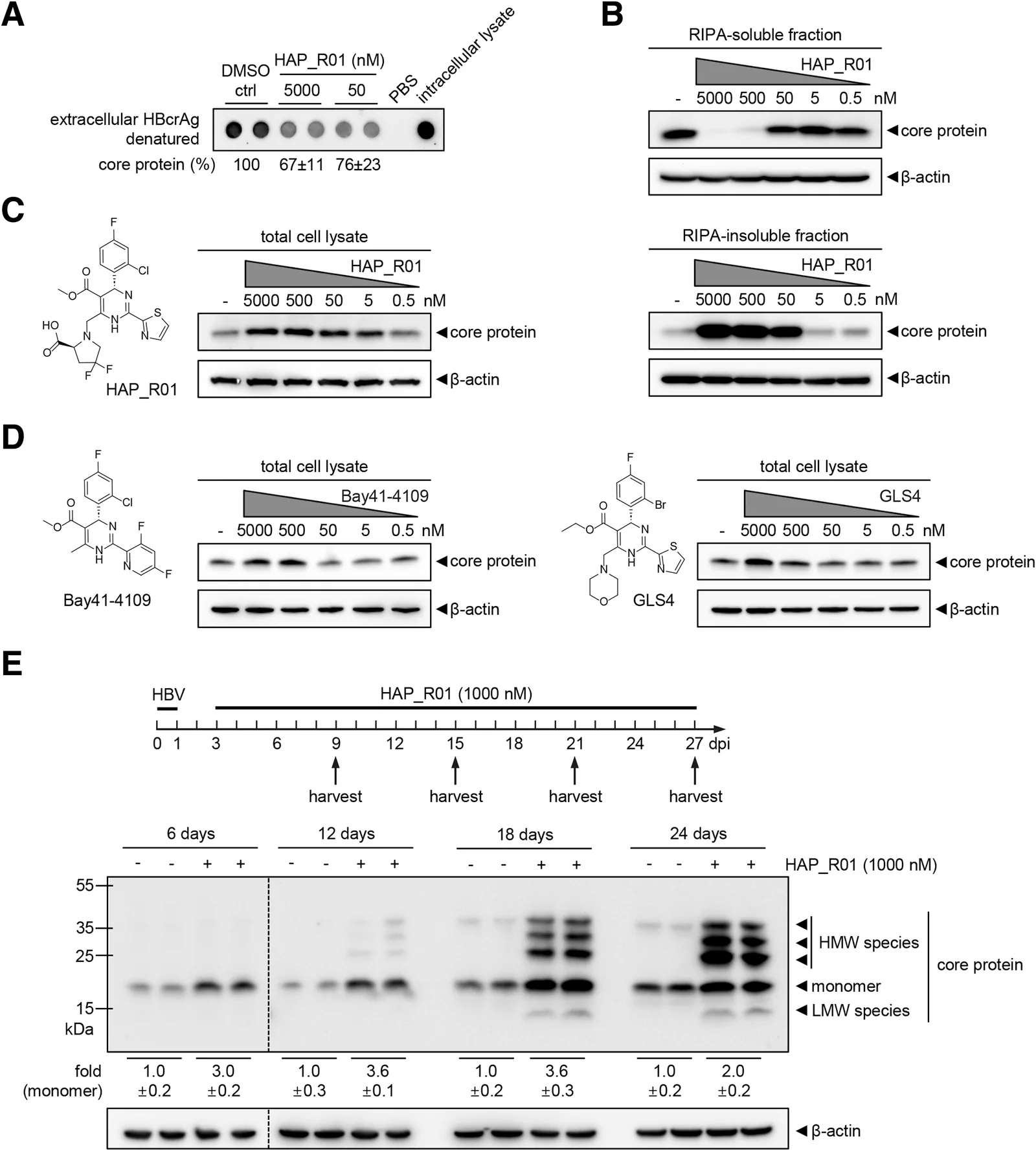
**Analysis of the fate of core protein upon CAM-A treatment.** (*A*) HBV-infected HepG2-NTCP-K7 cells were treated with HAP_R01 for 6 days at indicated concentrations. Extracellular media were collected and mixed with 1× Laemmli sample buffer and boiled for 10 minutes to analyze denatured core protein content by dot-blot. Representative images with densitometric quantification from 2 separate experiments are shown as mean ± standard deviation. (*B* and *C*) HepG2-NTCP-K7 cells were infected with HBV at a multiplicity of infection (moi) of 300 vp/cells, treated with decreasing concentrations of HAP_R01 for 6 days from day 3 PI on, and HBV core protein was analyzed by Western blot. β-actin serves as a loading control. (*B*) Cells were lysed with radioimmunoprecipitation assay (RIPA) buffer (containing 0.1% sodium dodecyl sulfate [SDS]) to obtain the RIPA-soluble fraction (*top*). The remaining pellets were then boiled with 1× Laemmli sample buffer to obtain the RIPA-insoluble fraction (*bottom*). (*C*) Cells were trypsinized, directly mixed with 1× Laemmli sample buffer, and boiled to obtain the total cell lysate. (*D*) Total cell lysate obtained after Bay41-4109 or GLS4 treatment was analyzed as described in panel (*C*). (*E*) HBV-infected HepG2-NTCP-K7 cells were treated with HAP_R01 (1000 nM) as indicated and harvested after 6, 12, 18, and 24 days of treatment. Total intracellular HBV core protein was analyzed by Western blotting. Monomeric core protein bands were quantified and are presented as mean ± standard deviation. HMW, high molecular weight; LMW, low molecular weight; vp, enveloped DNA containing virus particles.

Because the spherical and punctuate nuclear staining pattern of the core protein foci formed after HAP_R01 treatment resembled that of PML-NBs,^30^ we analyzed whether core protein is redistributed to PML-NBs. We observed core protein colocalizing with PML and Sp100, 2 essential components of PML-NBs, as evidenced by significantly higher Pearson correlation coefficient scores (Figure 9*A* and *B*).

**Figure 9:**
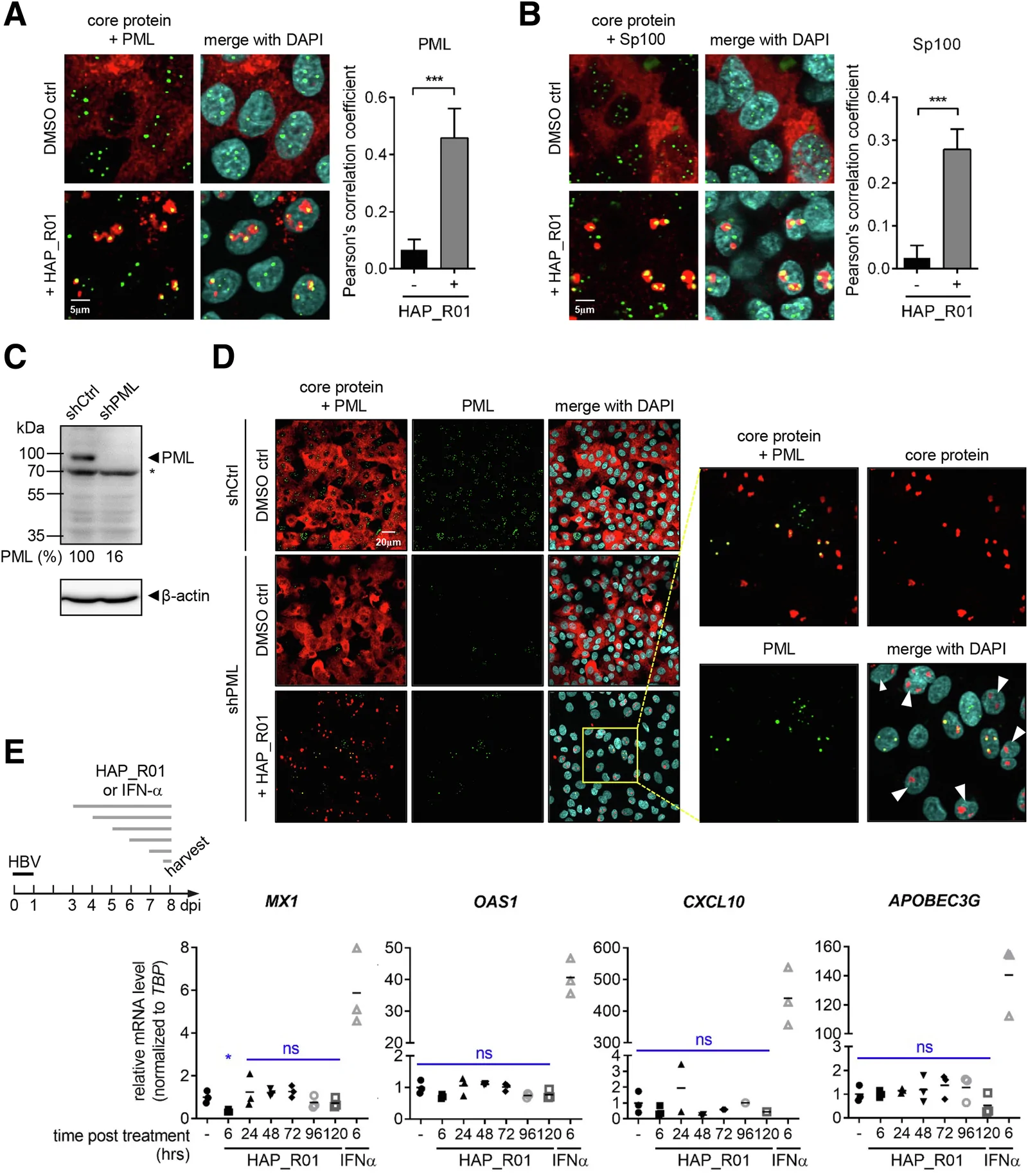
**Colocalization analysis of HBV core protein with PML-NBs following HAP_R01 treatment.** HBV-infected cells were treated with either DMSO or HAP_R01 (5 μM). HBV core protein together with PML (*A*) or Sp100 (*B*) were visualized by immunofluorescence staining. Colocalization between core protein and PML or Sp100 was evaluated by the Pearson correlation coefficient across multiple microscopic fields. Statistical significance was determined using the Student *t* test (∗∗∗*P* ≤.001). (*C*) HepG2-NTCP cells expressing either short hairpin (sh)Ctrl or shPML were lysed in radioimmunoprecipitation assay (RIPA) buffer, and PML expression was analyzed by Western blotting using an anti-PML antibody. An asterisk (∗) indicates a nonspecific band. β-actin serves as a loading control. Densitometric quantification of PML bands (relative to shCtrl) is shown below the blots. (*D*) HepG2-NTCP-shCtrl and HepG2-NTCP-shPML cells were infected with HBV at a multiplicity of infection (moi) of 500 vp/mL and treated with or without HAP_R01 (5 μM). HBV core protein and PML were visualized by immunofluorescence staining. Magnified views of the boxed subregion are shown. HBV core protein–positive but PML-depleted nuclei are indicated by *white arrowheads*. Nuclei were stained with 4′,6-diamidino-2-phenylindole (DAPI). (*E*) HepG2-NTCP-K7 cells were infected with HBV at an moi of 100 vp/cells and treated with HAP_R01 (5 μM) for the indicated time periods from 3 dpi. Interferon (IFN)-α (1500 IU/mL)-treated cells for 6 hours were included as an assay control. At 8 days PI, cells were harvested, and qRT-PCR was performed for selected genes. Relative messenger RNA (mRNA) expression normalized to TATA box binding protein (TBP) mRNA levels is shown. Statistical significance was determined using the Student *t* test (∗*P* ≤ .05, ns: not significant). vp, enveloped DNA containing virus particles.

To determine if PML itself has a role in formation of the core protein–specific foci, we generated HepG2-NTCP-K7 cells expressing PML short hairpin RNA (ie, HepG2-NTCP-K7-shPML cell population). Although PML was silenced in HBV-infected cells with more than 80% knockdown efficiency (Figure 9*C*), HAP_R01 still led to nuclear core protein aggregates (Figure 9*D*), indicating a minimal role, if any, of the PML protein. In addition, no induction of interferon-stimulated genes was observed, indicating that core protein aggregates do not trigger an innate immune response under our experimental conditions (Figure 9*E*).

Taken together, these results indicate that HAP_R01 induces nuclear relocalization of core protein to PML-NBs in a PML-independent manner with minimal contributions to core protein degradation and innate immune activation.

### HAP_R01 Preferentially Targets Newly Synthesized Core Protein

To get an insight of which form of core protein is targeted by HAP_R01, we assessed the turnover rate of cytoplasmic soluble core protein in HBV-infected HepG2-NTCP cells by pulse-chase experiments. Culturing cells with ^35^S-labeled methionine and cysteine during the pulse period and monitoring core protein levels over time during the subsequent chase allowed us to measure the stability of newly synthesized core protein (Figure 10*A*). In the absence of HAP_R01, core protein levels gradually increased for 1 hour and remained constant thereafter for 8 hours. Using HAP_R01, in contrast, resulted in a rapid decline of newly translated core protein with an estimated half-life of 1 hour. The initial slight increase is most likely due to continuous core protein translation using the remaining ^35^S-labeled amino acids within the cells.

**Figure 10:**
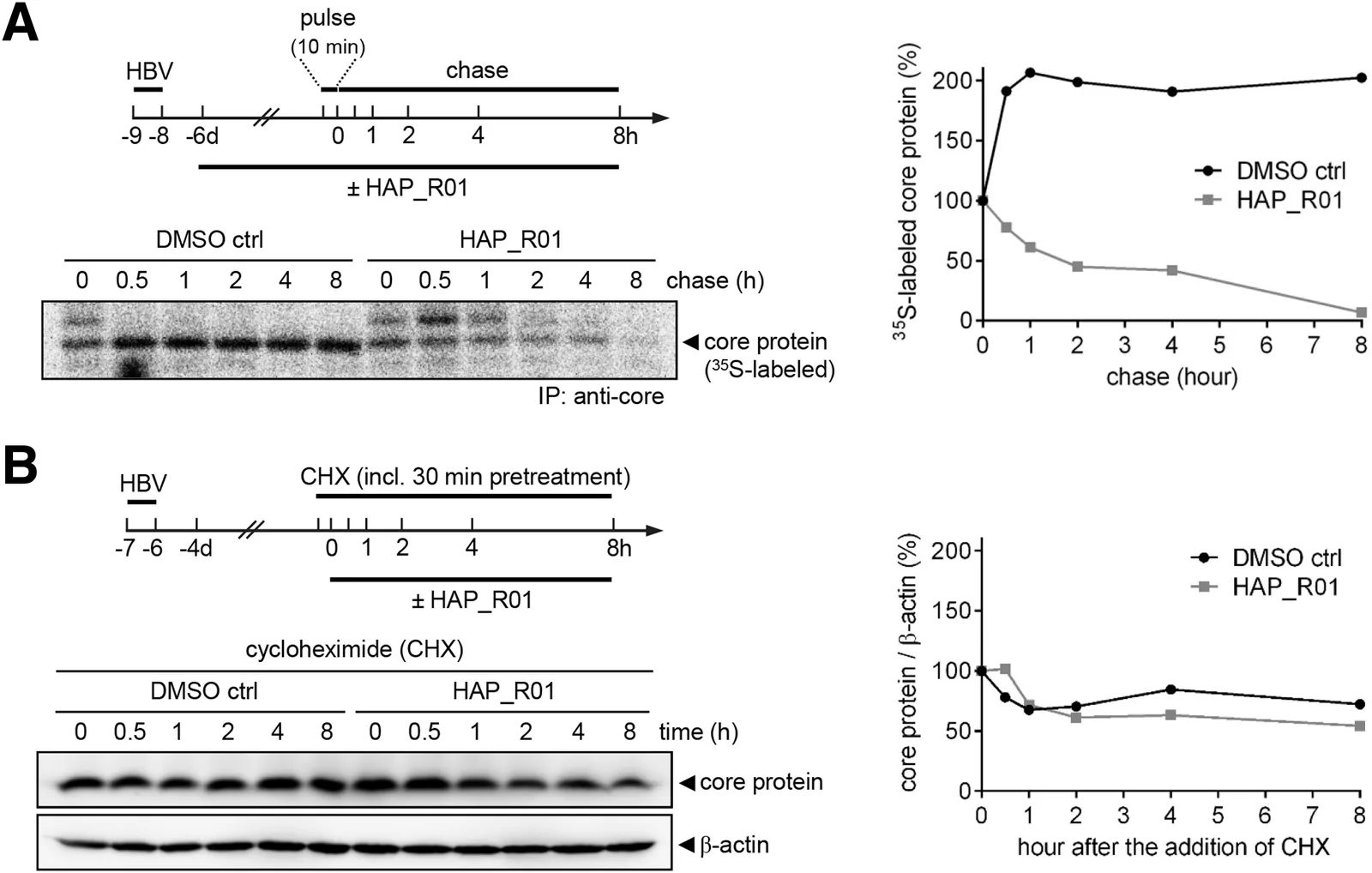
**Differential analysis of newly translated and preexisting core protein-targeting by HAP_R01.** (*A*) HepG2-NTCP-K7 cells were infected with HBV at a multiplicity of infection (moi) of 300 vp/cell and treated with HAP_R01 (5 μM) for 6 days. Cells were subjected to a 10-minute metabolic labeling (pulse) with [^35^S]-Cys and [^35^S]-Met and harvested for immunoprecipitation with an anti-core antibody at the indicated chase times (without [^35^S]). Autoradiogram of ^35^S-labeled core protein resolved by sodium dodecyl sulfate (SDS), SDS–polyacrylamide gel electrophoresis (PAGE) are shown. Quantitative analysis of ^35^S-labeled core protein is shown relative to time 0. (*B*) HBV-infected HepG2-NTCP-K7 cells were pretreated with CHX for 30 minutes before HAP_R01 (5 μM) was added for 8 hours. At indicated timepoints, cells were harvested and core protein levels were analyzed by Western blot. β-actin served as a loading control. Quantitative analysis of core protein normalized to β-actin is shown relative to time 0 (start of treatment). DAPI, 4′,6-diamidino-2-phenylindole; dpi, day postinfection; vp, enveloped DNA containing virus particles.

In an analogous experiment, the stability of pre-existing core protein, most likely assembled into capsid, was determined by pretreating cells with cycloheximide (CHX) 30 minutes before HAP_R01 treatment (Figure 10*B*). Upon CHX treatment, the cellular translation process is halted; therefore, we could measure the half-life of already mature proteins. We observed an approximately 27% reduction of soluble core protein levels in DMSO-treated cells during the 8-hour treatment, confirming the long half-life of core protein (>24 h).^18^ Adding HAP_R01 to CHX-pretreated cells caused a modest decrease in core protein levels (approximately 45%) during the 8-hour treatment period, indicating preferential recognition of newly synthesized core protein.

### HAP_R01 Reduces Covalently Closed Circular DNA and Hepatitis B Surface Antigen Levels During Long-Term Infection

Because HAP_R01 efficiently inhibited HBV replication in vivo and in cell culture but did not significantly affect intrahepatic cccDNA levels after a short, 2-week administration in HBV-infected mice (Figure 2*B*), we wondered if prolonged HAP_R01 treatment could affect established cccDNA pools. To explore this, we used HepG2-NTCP-K7 cells supporting cccDNA replenishment by intracellular genome recycling and de novo secondary infection.^6^ We treated HBV-infected cells with 1 μM HAP_R01 or ETV as a control from day 3 postinfection (PI) for 31 days (Figure 11*A*). As expected, both antivirals did not cause cytotoxicity (Figure 11*B*) and reduced total intracellular HBV-DNA levels by more than 3-log at day 21 PI (Figure 11C). In untreated cells, cccDNA levels increased by 2-fold until day 34 PI, whereas cells treated with HAP_R01 and ETV showed a significant decrease in cccDNA levels from day 21 PI onwards (Figure 11*D*), with HAP_R01 exerting a faster and stronger effect. Accordingly, secreted HBsAg and HBeAg gradually increased over time if cells remained untreated (Figure 11*E* and *F*). In treated cells, HBsAg and HBeAg reached a plateau at day 12 PI and slowly decreased thereafter under HAP_R01 and ETV treatment. Overall, our results demonstrated that long-term administration of HAP_R01 can reduce established cccDNA pool and HBsAg secretion.

**Figure 11:**
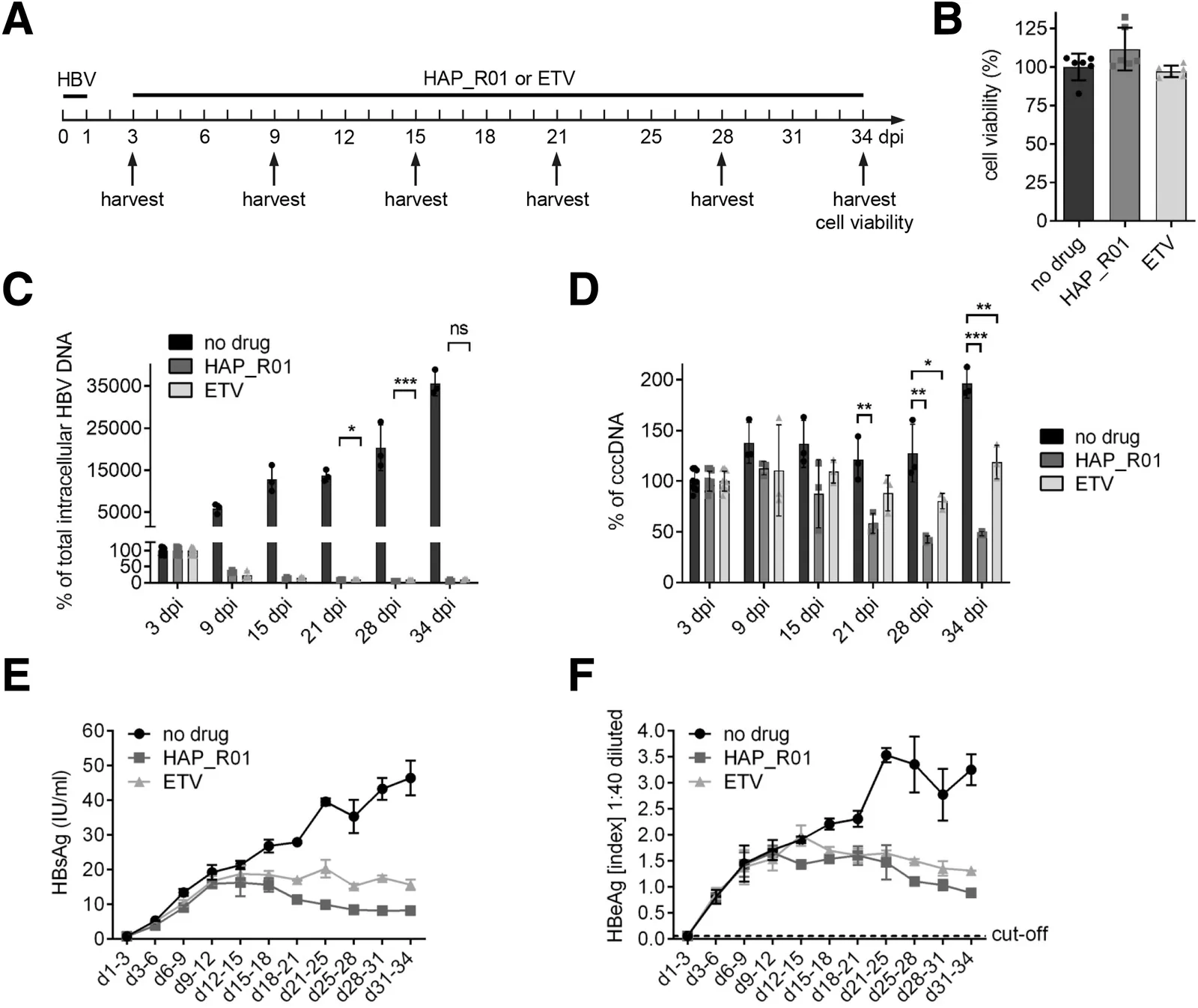
**Effect of long-term HAP_R01 treatment on viral parameters.** (*A*) Experimental setup. HepG2-NTCP-K7 cells were infected with HBV at a multiplicity of infection (moi) of 100 vp/cells and either left untreated or treated with HAP_R01 (1 μM) or ETV (1 μM). Cells were maintained in the presence of antivirals and harvested at indicated timepoints. (*B*) At 34 dpi, cell viability was assessed by CellTiter-Blue assay (*C* and *D*) Total intracellular HBV-DNA and cccDNA levels were analyzed by qPCR. For cccDNA detection, extracted total cellular DNA was treated with T5 exonuclease prior to qPCR reaction. Given percentage values were normalized to *PRNP* and *MT (mitochondrial)-CO3* genes and relative to respective HBV-DNA levels at 3 dpi. (*E* and *F*) HBsAg and HBeAg were quantified using a commercial immunoassay. Statistical significance was determined by the Student *t* test (∗∗∗*P* ≤.001, ∗∗*P* ≤.01, ∗*P* ≤.05, ns: not significant). dpi, day postinfection; vp, enveloped DNA containing virus particles.

## Discussion

In this study, we demonstrated that the potent CAM-A, HAP_R01, inhibits viral DNA replication and cccDNA replenishment by promoting empty capsid formation primarily targeting newly translated core protein, redistributing core protein to the nucleus and inducing an insoluble agglomerate of core protein at PML-NBs. Over time, this results in a loss of cccDNA in treated cells and a reduction of secreted HBsAg.

HAP_R01 has several advantages as a new anti-HBV drug candidate. First, HAP_R01 inhibited viral genome replication originating from NUC-resistant HBV mutants at comparable levels to that from a WT HBV genome. Second, the effect of HAP_R01 is pan-genotypic. Third, HAP_R01 showed a sustained suppression of viral DNA synthesis for 2 weeks after treatment cessation, with an only slow rebound of core protein, indicating a reversible and core protein–specific antiviral effect. Lastly, HAP_R01 exhibited 2-digit nanomolar EC_50_ (20.5–39.9 nM) with a >100 μM half-maximal cytotoxic concentration (CC_50_) value,^24^ yielding a therapeutic index (CC_50_/EC_50_) of >2500. These results highlighted the drug-like properties of HAP_R01 and encouraged us to evaluate its antiviral activity in vivo.

A human liver chimeric uPA/SCID mouse model was chosen because repopulated human hepatocytes support de novo HBV infection and show both cytoplasmic and nuclear distribution of intrahepatic core protein, allowing us to monitor drug-induced changes in its level and subcellular localization. Interestingly, in addition to 1.3- to 2.7-log reductions in serum HBV-DNA levels, 2-week oral administration of HAP_R01 resulted in decreased cytoplasmic and increased nuclear core protein staining. To the best of our knowledge, this is the first report describing the role of CAM-A compounds in the subcellular localization changes of core protein in vivo. Previous studies have showed a decrease in cytoplasmic core protein levels following treatment with other CAM-A compounds, such as Bay41-4109 administration in HBV transgenic mice^31^ and GLS4 administration in nude mice subcutaneously inoculated with HepAD38 cells.^16^ Additionally, a lower frequency of HBV core protein–positive cells was observed in AAV-HBV–transduced mice after treatment with RG7907 or HAP-1.^21^^,^^22^ However, nuclear redistribution of core protein was not noted, possibly due to the strong basal nuclear core protein staining in these mice.

We demonstrated that HAP_R01 exhibits distinct antiviral activity depending on concentration. At 50 nM (2 times the EC_50_), HAP_R01 treatment induced the assembly of slower migrating empty capsids that increased, correlating with core protein levels, in the cytoplasm and even more in the nucleus. This finding implies that, at a concentration where the drug binding sites are not yet saturated, HAP_R01 may stabilize newly forming capsids. However, in line with in vitro observations^15^^,^^32^ and similar to phenylpropenamides and SBAs, HAP_R01 may also accelerate the formation of structurally altered empty capsids, which could explain the slightly higher signals for capsids than core protein during HAP_R01 treatment (Figure 7*A*). Such accelerated assembly could be too fast for proper encapsidation of pgRNA and HBV polymerase, or the altered capsids do not favor incorporation of pgRNA-HBV polymerase complex at all.

It is noteworthy that the levels of core protein were gradually increasing in the nucleus during 50 nM HAP_R01 treatment. This is consistent with our in vivo observations, highlighting the physiological relevance of our findings. Because HBV replicates in the cytoplasm, nuclear redistribution of core protein can be considered as a novel antiviral mechanism of CAMs. Given that core protein contains nuclear localization signals and nuclear export signals,^28^^,^^29^ further studies of the role of these regulatory motifs would help in elucidating how HAP_R01 regulates core protein redistribution.

At higher concentrations, HAP_R01 preferentially targeted newly synthesized core protein, leading to its condensation within PML-NBs. This observation is in line with previous studies reporting the crystal structure of the core protein Y132A-HAP_R01 complex and showing colocalization of HBV core protein with PML protein after treatment with other HAP derivatives (Bay38-7690 and RG7907).^15^^,^^33^^,^^34^ It is plausible that HAP_R01 binds to freshly translated core protein dimers, leading to kinetically trapped assembly intermediates that readily translocate into the nucleus where they accumulate and aggregate. HAP_R01-mediated deposition of insoluble core protein at PML-NBs remains an open question in terms of both mechanism and underlying determinants. Because nuclear core protein foci were still visible in PML-depleted cells, we reason that PML itself may not be the primary factor guiding core protein recruitment, but rather that another component of PML-NBs may mediate this localization. Given that many proteins residing in PML-NBs are SUMOylated and that HBV core protein can undergo SUMO modification at lysine residues within putative SUMO conjugation motifs,^35^ it is possible that PML-NBs serve as a scaffold for interactions between cellular and viral proteins. In this context, HAP_R01-induced nuclear redistribution of core protein may further promote its accumulation within PML-NBs. At present, it remains unclear whether recruitment to PML-NBs precedes core protein insolubilization or whether aggregation itself drives subsequent sequestration to these nuclear structures. Further in-depth investigations are required to determine whether HBV core protein undergoes SUMOylation and, if so, whether this modification is enhanced following CAM-A treatment and to identify host factors that potentially coaggregate with the core protein, as suggested by the appearance of high-molecular–weight core protein species observed exclusively after HAP_R01 treatment.

Previous studies defined core protein depletion, potentially via proteasomal or lysosomal degradation, as the primary antiviral effect of HAP-type CAMs in cell-culture.^15^^,^^16^^,^^18^^,^^19^^,^^33^ None of these studies, however, analyzed core protein contents in the insoluble cellular fraction. This, together with the redistribution of cytoplasmic core protein to the nucleus and a modest increase in the rate of core protein degradation, likely led to a misinterpretation of HAP’s mechanism-of-action. In this study, we demonstrated that total core protein levels (soluble core protein plus insoluble core protein) were not reduced and even increased upon HAP_R01 treatment. Importantly, this was also the case for Bay41-4109 and GLS4, indicating that intracellular degradation of core protein is not the main mode-of-action of HAP compounds. Our finding highlights that CAM-A primarily induces core protein accumulation in PML-NBs where it resides in insoluble forms and is stabilized.

cccDNA persists in chronically infected human liver. Given that HBV maintains stable cccDNA pools by de novo secondary infection and intracellular recycling of HBV genomes,^6^ we sought to examine whether HAP_R01 is able to inhibit cccDNA replenishment. As expected, in long-term cultures, cccDNA levels increased by 2-fold in the untreated cells, whereas 31-day treatment of ETV blocked the cccDNA increase by inhibiting cccDNA replenishment from intracellular, HBV-DNA–containing capsids.^6^ Continuous HAP_R01 treatment even lowered the cccDNA levels below the initially established cccDNA level. This may be explained by a stronger inhibition of the formation of DNA-containing capsids and HBV replication during long-term treatment or by an additional effect of HAP_R01 (eg, by affecting hepatocyte physiology through protein accumulation). It remains premature, however, to directly compare the antiviral potency of CAMs and NUCs based on our current findings in NTCP-reconstituted HepG2 cells. We did not observe an induction of interferon-stimulated genes as a sign of an activation of pattern recognition pathways, although an immune response to the core protein aggregates cannot be excluded.

Kum et al recently reported that RG7907 treatment induces hepatocyte death and compensatory proliferation, accompanied by transient upregulation of genes involved in apoptosis, mitosis, and interferon pathways,^21^ which was not observed in our study. This discrepancy could be attributed to differences in experimental models or the level of intracellular core protein (physiological level vs unphysiological level).^22^^,^^23^ Additionally, the treatment duration may play a role, as evidenced by the formation of larger nuclear core protein aggregates in fewer cells over time following HAP_R01 treatment. We speculate that high levels of core protein could result in significant accumulation of core protein aggregates in CAM-A–treated cells. If these insoluble core protein matters cannot be properly cleared and surpass a critical threshold, they may induce cellular stress and ultimately trigger cell death. Given that additional immunologic effects beyond their direct antiviral activity were recently reported for the CAM-E GLP-26,^36^ more sophisticated in vivo studies are warranted to determine whether CAM-A–mediated apoptotic cell death and/or immune responses also occur in the clinical setting or remain restricted to preclinical models.

In summary, our study provides deep mechanistic insights into CAM-A compounds and highlights their strong antiviral effects on capsid assembly and core protein distribution and solubility, as well as cccDNA replenishment. We previously demonstrated that HAP_R01 can target capsids of incoming HBV particles and thus interfere with establishment of HBV infection^24^ and can inhibit HBeAg biosynthesis.^25^ These observations suggest that CAMs exert pleiotropic antiviral effects across multiple stages of the HBV life cycle and support their further clinical evaluation as monotherapy or in combination with antivirals with distinct mechanisms of action or immunomodulatory strategies.

## Materials and Methods

### Cell Culture and Hepatitis B Virus Infection

HepG2, HepG2-derived cells, and Huh7-NTCP cells were cultured in Dulbecco’s Modified Eagles Medium supplemented with 10% fetal bovine serum, 100 U/mL penicillin–streptomycin, 2 mM L-glutamine, 1 mM sodium pyruvate, and 1× nonessential amino acids (all from Gibco, Thermo Fisher Scientific). Cell-culture derived HBV (genotype D; ayw) was prepared and used for infection studies as previously described.^6^^,^^37^ Briefly, the virus-containing supernatant from HepAD38 cells were collected and concentrated using heparin columns to produce infectious HBV particles. HepG2-NTCP-K7 cells were inoculated with HBV at an indicated multiplicity of infection in the presence of 4% PEG6000 for 16 to 24 hours. The HBV inoculum was removed, and the cells were maintained in regular media containing 2.5% DMSO. Primary human hepatocytes were prepared and infected with HBV as described elsewhere.^38^ Quantitiative real-time polymerase chain reaction (qRT-PCR) analysis of cccDNA and total intracellular HBV-DNA were performed as described previously.^6^ HAP_R01 was synthesized by Roche Innovation Center Shanghai as described previously.^39^ Bay41-4109 and AT130 were kindly provided by Janssen Pharmaceutica. All other drugs were purchased from Sigma-Aldrich.

### Plasmid Transfection

The following replication-competent HBV plasmids were used to induce intracellular HBV replication: pCH-9_3091,^40^ pTHBV1.3WT (genotype D), pRVHBV1.5_A (genotype A; kindly provided by Volker Bruss), pZDonor_N4214_B2 and pZDonor_N3825_C1 (genotype B and C, respectively; kindly provided by Dieter Glebe), pCH-9_3091 plasmid harboring rtL180M/rtM204V or rtI169T/rtL180M/rtM204V/rtM250V mutations in the RT domain of the HBV polymerase. pCMV-HBVcore plasmid expressing HBV core protein (genotype D) was a gift from Wang-Shick Ryu. Fugene HD transfection reagent (Promega) or linear polyethylenimine (Polysciences) was used for transfecting HepG2 cells.^24^^,^^41^

### Southern Blot Analysis of Hepatitis B Virus Capsid-Associated DNA

Cells were lysed with 50 mM Tris-HCl (pH 8.0), 150 mM NaCl, 1 mM EDTA, and 1% NP-40 for 15 minutes on ice and were clarified by centrifugation. The clarified cell lysate was treated with 20 μg/mL DNase I and 200 μg/mL RNase A (Sigma-Aldrich) in the presence of 10 mM MgCl_2_ at 37 °C for 3 hours. After centrifugation, cytoplasmic capsids were precipitated with 8.8% PEG8000 and resuspended in 10 mM Tris-HCl (pH 7.5), 150 mM NaCl, and 1 mM EDTA. This capsid preparation was digested with 0.5% sodium dodecyl sulfate and 240 μg/mL proteinase K (Carl Roth), followed by phenol-chloroform extraction and ethanol precipitation of the extract viral DNAs. Alternatively, capsid-associated DNA was isolated by column purification using the QIAamp MinElute Virus Spin Kit (Qiagen). Viral DNAs were electrophoresed through an agarose gel and transferred onto a nylon membrane. After ultraviolet cross-linking, the membrane was hybridized with a digoxigenin-labeled HBV-specific DNA probe.^42^

### Human Liver Chimeric Mice Procedures

Human liver chimeric uPA/SCID mice were generated by PhoenixBio Co, Ltd (Higashi). Mice were inoculated with 10^6^ copies of HBV genotype C (accession no. AB246345) via the retro-orbital route. Eight weeks post infection, 16 male mice with serum HBV-DNA levels above 10^6^ copies/mL were randomized into 4 groups based on body weight, blood human albumin concentration, and serum HBV-DNA level. The groups of 4 mice received either vehicle control twice daily, 3 or 10 mg/kg of HAP_R01 twice daily, or 5 mg/kg of TDF once daily by oral gavage for 2 weeks. The handling and experimental procedures using the animals for this study was approved by the Animal Ethics Committee of PhoenixBio and adhered to the Guide for the Care and Use of Laboratory Animals.

Serum samples were collected every 2 to 3 days for the measurements of HBV markers and human albumin. Blood human albumin concentration was determined by latex agglutination immunonephelometry (LX Reagent “Eiken” Alb II, Eiken Chemical Co, Ltd) using the microplate reader system Vmax (Molecular Device Corporation). Serum HBV DNA was extracted using the SMITEST EX-R&D Nucleic Acid Extraction Kit (Medical & Biological Laboratories Co, Ltd). The qRT-PCR was performed using the TaqMan PCR Core Reagents and ABI Prism 7500 sequence detector system (Life Technologies Corporation). The initial activation of uracil-N-glycosylase at 50 °C for 2 minutes was followed by the inactivation of uracil-N-glycosylase at 95 °C for 10 minutes. Subsequent PCR amplification consisted of 53 cycles of denaturation at 95°C for 20 seconds and annealing and extension at 60 °C for 1 minute per cycle. The primers and probe consisted of forward primer, 5′-CACATCAGGATTCCTAGGACC-3′; reverse primer, 5′-AGGTTGGTGAGTGATTGGAG-3′; and TaqMan probe, 5′-FAM-CAGAGTCTAGACTCGTGGTGGACTTC-TAMRA-3′. The lowest quantification limit of the assay was 8.0 × 10^3^ copies/mL serum. Serum HBsAg and HBeAg concentrations were determined by SRL, Inc based on chemiluminescence enzyme immunoassays developed by Abbott (Architect System). The lowest quantification limits of the assays were 1.5 IU/mL serum and 15 S/CO, respectively.

At the end of treatment, all animals were sacrificed, and the livers were harvested for intrahepatic HBV core protein detection. Two slices of 3- to 5-mm in thickness were cut from the left lateral lobe of the mouse liver, fixed with 10% neutral-buffered formalin, and embedded in paraffin. The sectioned paraffin-embedded blocks were mounted on 3-aminopropyltriethoxysilane-coated slide glass. Immunohistochemical staining of HBV-infected hepatocytes was using a polyclonal rabbit antibody against core protein (anti-core, DAKO) and the SuperPicture Polymer Detection kit (Invitrogen).

### Cell Fractionation and Analysis of Hepatitis B Virus Proteins and Capsids

Separation of the nuclear and cytoplasmic fractions was carried out using the NE-PER Nuclear and Cytoplasmic Extraction Reagent (Thermo Fisher Scientific). Cytoplasmic and nuclear extracts were directly loaded onto the agarose gel for native agarose gel electrophoresis^24^ or analyzed by Western blot. The remaining pellets after subcellular fractionation were resuspended in 1× Laemmli sample buffer and boiled at 95 °C for 10 minutes to yield an insoluble fraction of cell lysate. Western blotting was performed as described previously with the following primary antibodies^6^: anti-core mouse hybridoma supernatant (in-house 8C9; 1:7), polyclonal rabbit anti-core serum (H800, 1:400), polyclonal anti-LHBs rabbit serum (H863, gift from Stephan Urban, 1:1000), anti–β-actin (Sigma-Aldrich, 1:5000), anti–α-tubulin (Sigma-Aldrich, 1:3000), anti-Lamin A/C (BD Bioscience, 1:000), and anti-GAPDH (Acris, 1:3000). Both 8C9 and H800 preferentially detect core protein under denaturing conditions.

### Northern Blot Analysis of Hepatitis B Virus RNAs

Northern blot analysis of HBV RNAs was performed as described previously.^6^^,^^43^

### Southern Blot Analysis of Covalently Closed Circular DNA

Southern blot analysis of cccDNA was performed as described previously.^24^

### Cesium Chloride Density Gradient Centrifugation and Dot Blot Analysis of Hepatitis B Virus Capsid

Cell lysate obtained from HepG2-NTCP-K7-H1.3Lˉ cells was subjected to cesium chloride density gradient ultracentrifugation followed by dot blot analysis, as described previously.^6^^,^^24^

### Immunofluorescence Analysis of Viral and Host Proteins

Immunofluorescence analyses were performed as described previously with the following primary antibodies^6^: polyclonal rabbit anti-core sera (DAKO, 1:400; Ad53, 1:400; Cell Marque,1:200); anti-PML (SantaCruz, 1:100); and anti-Sp100 (Millipore, 1:200). Due to limited antibody availability, different anti-core sera—primarily recognizing capsid-associated core protein—were used in separate experiments, and comparable results were obtained across different antibodies. Images were collected either by a confocal laser scanning microscope (Fluoview FV10i, Olympus) or a spinning disc microscope (Nikon Eclipse Ti, Perkin Elmer). The Pearson correlation coefficient was determined by the Volocity 6.2 software package (Perkin Elmer).

### Quantitative Reverse Transcription Polymerase Chain Reaction of Immune-Regulatory Genes

qRT-PCR was performed as described previously.^24^ Briefly, RNA extraction was carried out by using NucleoSpin RNA kit (Macherey-Nagel), and complementary DNA synthesis was performed with SuperScript III First-stand Synthesis System (Invitrogen). qRT-PCR was conducted using LightCycler480 SYBR Green I master mix and the LightCycler480 Instrument (Roche). Relative mRNA expression was normalized to TATA-binding protein. Primer sequences (5′–3′) were as follows: MX1 (Forward: TGAAGAACTGGATGATCAAAGG and Reverse: ACCTGATGGCCTATCACCAG), OAS1 (Forward: TGATGCCCTGGGTCAGTT and Reverse: TTCTTACAATTTTGGTACCAGTGC), CXCL10 (Forward: TATTCCTGCAAGCCAATTTTG and Reverse: TCTTGATGGCCTTCGATTCTG), APOBEC3G (Forward: CCGAGGACCCGAAGGTTAC and Reverse: TCCAACAGTGCTGAAATTCG), and TBP (Forward: TATAATCCCAAGCGGTTTGC and Reverse: CTGTTCTTCACTCTTGGCTCCT)

### Pulse-Chase Experiment

In vitro metabolic labeling of cellular proteins was performed by supplementing the culture media with ^35^S-Cys and ^35^S-Met at the final concentration of 0.1 mCi/mL each. After 10 minutes, cells were washed with phosphate-buffered saline twice and further incubated in medium containing unlabeled amino acids for the indicated chase periods. Core proteins were immunoprecipitated using an anti-core antibody (DAKO) and analyzed by sodium dodecyl sulfate–polyacrylamide gel electrophoresis. Radioactive signals were detected using a phosphor imaging system (CR 35 Bio, Raytest Isotopenmessgeraete GmbH)

### Cell Viability Assay

Cell viability was analyzed using CellTiter-Blue reagent (Promega). Briefly, confluent cells were incubated with a 1:5 mixture of CellTiter-Blue and culture medium for 1 hour until the mixture turned a purple or pink color. Fluorescence was measured using a Tecan Infinite F200 microplate reader (Tecan) at excitation and emission wavelengths of 560 nm and 590 nm, respectively.

### Hepatitis B Surface Antigen and Hepatitis B e Antigen Measurement

Extracellular HBsAg and HBeAg levels from cell culture–derived samples were quantified on an Architect platform using the quantitative HBsAg test (Reference, 6C36-44; Cutoff, 0.25 IU/mL). HBeAg levels were analyzed by qualitative test on an automated BEP III system (Siemens).

### Statistical Analysis

All data were presented as the mean ± standard deviation unless otherwise stated. Differences between groups were analyzed using the Student *t* test. The Pearson correlation coefficient was used to test the linear correlation. *P* values ≤ .05 were considered statistically significant (∗∗∗*P* ≤ .001, ∗∗*P* ≤ .01, ∗*P* ≤ .05, ns: not significant). Statistical analyses were conducted using GraphPad Prism version 9.

## Supplementary Material

Supplementary Material

Extended PDF

## References

[1] Seeger C., Mason W.S. (2015). Molecular biology of hepatitis B virus infection. Virology.

[2] Thomas D.L. (2019). Global Elimination of Chronic Hepatitis. N Engl J Med.

[3] WHO (2025). Hepatitis B, Fact sheet. https://www.who.int/news-room/fact-sheets/detail/hepatitis-b.

[4] Stanaway J.D., Flaxman A.D., Naghavi M. (2016). The global burden of viral hepatitis from 1990 to 2013: findings from the Global Burden of Disease Study 2013. Lancet.

[5] Nassal M. (2015). HBV cccDNA: viral persistence reservoir and key obstacle for a cure of chronic hepatitis B. Gut.

[6] Ko C., Chakraborty A., Chou W.M. (2018). Hepatitis B virus genome recycling and de novo secondary infection events maintain stable cccDNA levels. J Hepatol.

[7] Zoulim F., Locarnini S. (2009). Hepatitis B virus resistance to nucleos(t)ide analogues. Gastroenterology.

[8] Fanning G.C., Zoulim F., Hou J. (2019). Therapeutic strategies for hepatitis B virus infection: towards a cure. Nat Rev Drug Discov.

[9] Niklasch M., Zimmermann P., Nassal M. (2021). The hepatitis B virus nucleocapsid-dynamic compartment for infectious virus production and new antiviral target. Biomedicines.

[10] Kim H., Ko C., Lee J.Y. (2021). Current progress in the development of hepatitis B virus capsid assembly modulators: chemical structure, mode-of-action and efficacy. Molecules.

[11] Segal-Maurer S., DeJesus E., Stellbrink H.J. (2022). Capsid inhibition with lenacapavir in multidrug-resistant HIV-1 infection. N Engl J Med.

[12] Zoulim F., Zlotnick A., Buchholz S. (2022). Nomenclature of HBV core protein-targeting antivirals. Nat Rev Gastroenterol Hepatol.

[13] Feld J.J., Colledge D., Sozzi V. (2007). The phenylpropenamide derivative AT-130 blocks HBV replication at the level of viral RNA packaging. Antiviral Res.

[14] Campagna M.R., Liu F., Mao R. (2013). Sulfamoylbenzamide derivatives inhibit the assembly of hepatitis B virus nucleocapsids. J Virol.

[15] Zhou Z., Hu T., Zhou X. (2017). Heteroaryldihydropyrimidine (HAP) and Sulfamoylbenzamide (SBA) inhibit hepatitis B virus replication by different molecular mechanisms. Sci Rep.

[16] Wu G., Liu B., Zhang Y. (2013). Preclinical characterization of GLS4, an inhibitor of hepatitis B virus core particle assembly. Antimicrob Agents Chemother.

[17] Stray S.J., Zlotnick A. (2006). BAY 41-4109 has multiple effects on Hepatitis B virus capsid assembly. J Mol Recognit.

[18] Deres K., Schroder C.H., Paessens A. (2003). Inhibition of hepatitis B virus replication by drug-induced depletion of nucleocapsids. Science.

[19] Lin J., Yin L., Xu X.Z. (2022). Bay41-4109-induced aberrant polymers of hepatitis b capsid proteins are removed via STUB1-promoted p62-mediated macroautophagy. PLoS Pathog.

[20] Taverniti V., Meiss-Heydmann L., Gadenne C. (2024). CAM-A-dependent HBV core aggregation induces apoptosis through ANXA1. JHEP Rep.

[21] Kum D.B., Vanrusselt H., Acosta Sanchez A. (2023). Class A capsid assembly modulator RG7907 clears HBV-infected hepatocytes through core-dependent hepatocyte death and proliferation. Hepatology.

[22] Berke J.M., Tan Y., Sauviller S. (2024). Class A capsid assembly modulator apoptotic elimination of hepatocytes with high HBV core antigen level in vivo is dependent on de novo core protein translation. J Virol.

[23] Kornyeyev D., Song Z., Eng S. (2024). Selective depletion of HBV-infected hepatocytes by class A capsid assembly modulators requires high levels of intrahepatic HBV core protein. Antimicrob Agents Chemother.

[24] Ko C., Bester R., Zhou X. (2019). A new role for capsid assembly modulators to target mature hepatitis B virus capsids and prevent virus infection. Antimicrob Agents Chemother.

[25] Yan Z., Wu D., Hu H. (2019). Direct inhibition of hepatitis B e antigen by core protein allosteric modulator. Hepatology.

[26] Wu S., Luo Y., Viswanathan U. (2018). CpAMs induce assembly of HBV capsids with altered electrophoresis mobility: Implications for mechanism of inhibiting pgRNA packaging. Antiviral Res.

[27] Guidotti L.G., Matzke B., Schaller H. (1995). High-level hepatitis B virus replication in transgenic mice. J Virol.

[28] Yang C.C., Chang C.H., Chen H.L. (2022). CRM1-spike-mediated nuclear export of hepatitis B virus encapsidated viral RNA. Cell Rep.

[29] Li H.C., Huang E.Y., Su P.Y. (2010). Nuclear export and import of human hepatitis B virus capsid protein and particles. PLoS Pathog.

[30] Hsu K.S., Kao H.Y. (2018). PML: Regulation and multifaceted function beyond tumor suppression. Cell Biosci.

[31] Weber O., Schlemmer K.H., Hartmann E. (2002). Inhibition of human hepatitis B virus (HBV) by a novel non-nucleosidic compound in a transgenic mouse model. Antiviral Res.

[32] Stray S.J., Bourne C.R., Punna S. (2005). A heteroaryldihydropyrimidine activates and can misdirect hepatitis B virus capsid assembly. Proc Natl Acad Sci U S A.

[33] Huber A.D., Wolf J.J., Liu D. (2018). The Heteroaryldihydropyrimidine Bay 38-7690 Induces Hepatitis B Virus Core Protein Aggregates Associated with Promyelocytic Leukemia Nuclear Bodies in Infected Cells. mSphere.

[34] Vanrusselt H., Kum D.B., Taverniti V. (2023). Novel non-HAP class A HBV capsid assembly modulators have distinct in vitro and in vivo profiles. J Virol.

[35] Hofmann S., Plank V., Groitl P. (2023). SUMO modification of hepatitis B virus core mediates nuclear entry, promyelocytic leukemia nuclear body association, and efficient formation of covalently closed circular DNA. Microbiol Spectr.

[36] Fernandes P., Wang Y., Doisne J.M. (2026). Functional immune responses induced by a capsid assembly modulator in chronic hepatitis B virus-infected humanized mice. Cell Host Microbe.

[37] Wettengel J.M., Linden B., Esser K. (2021). Rapid and robust continuous purification of high-titer hepatitis B virus for in vitro and in vivo applications. Viruses.

[38] Kleine M., Riemer M., Krech T. (2014). Explanted diseased livers - a possible source of metabolic competent primary human hepatocytes. PLoS One.

[39] Qiu Z., Lin X., Zhang W. (2017). Discovery and pre-clinical characterization of third-generation 4-H Heteroaryldihydropyrimidine (HAP) analogues as Hepatitis B Virus (HBV) capsid inhibitors. J Med Chem.

[40] Nassal M. (1992). The arginine-rich domain of the hepatitis B virus core protein is required for pregenome encapsidation and productive viral positive-strand DNA synthesis but not for virus assembly. J Virol.

[41] Ko C., Shin Y.C., Park W.J. (2014). Residues Arg703, Asp777, and Arg781 of the RNase H domain of hepatitis B virus polymerase are critical for viral DNA synthesis. J Virol.

[42] Ko C., Su J., Festag J. (2021). Intramolecular recombination enables the formation of hepatitis B virus (HBV) cccDNA in mice after HBV genome transfer using recombinant AAV vectors. Antiviral Res.

[43] Ko C., Lee S., Windisch M.P. (2014). DDX3 DEAD-Box RNA helicase is a host factor that restricts hepatitis B virus replication at the transcriptional level. J Virol.

