# Supplementary material for: A New Role of Class A Hepatitis B Virus Capsid Assembly Modulators in Core Protein Dynamics and Covalently Closed Circular DNA Replenishment
Source: Cell Mol Gastroenterol Hepatol. 2026 Aug 12;20(11):101860. doi: 10.1016/j.jcmgh.2026.101860 (PMC13629346; doi:10.1016/j.jcmgh.2026.101860)

Original Western blot

(plus Southern blot, Northern blot, native agarose gel, and autoradiography original images)

Fig. 1C (Southern blot)

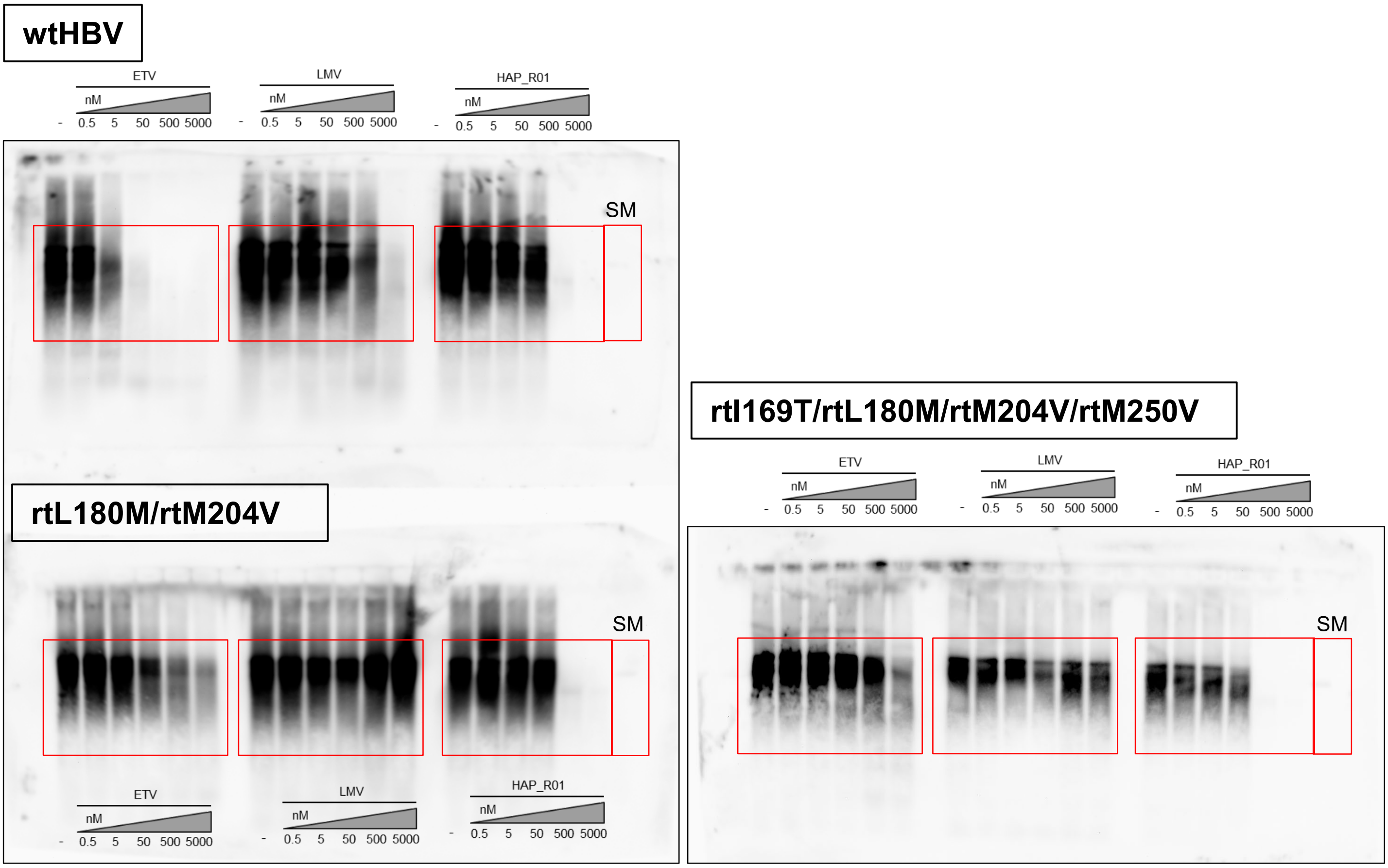

intracellular capsid-associated HBV DNA (rcDNA+dlDNA+ssDNA)

Fig. 1D (Southern blot)

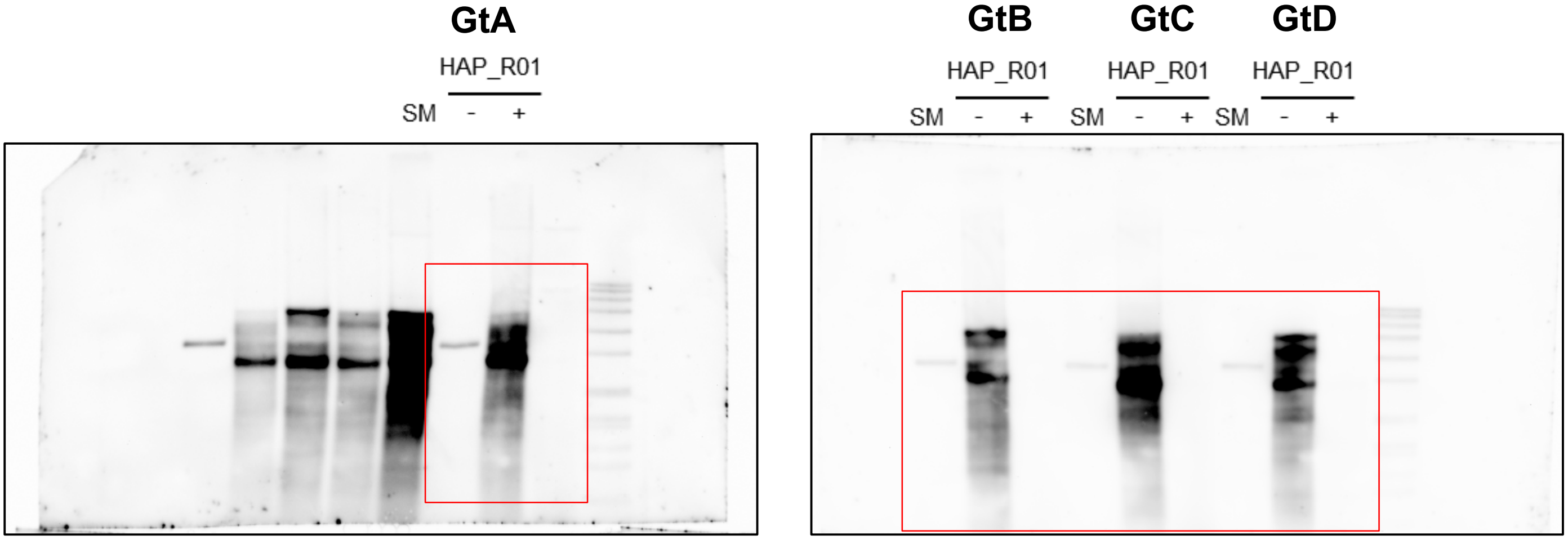

intracellular capsid-associated HBV DNA (rcDNA+dlDNA+ssDNA)

Fig. 3B (Southern blot + Western blot)

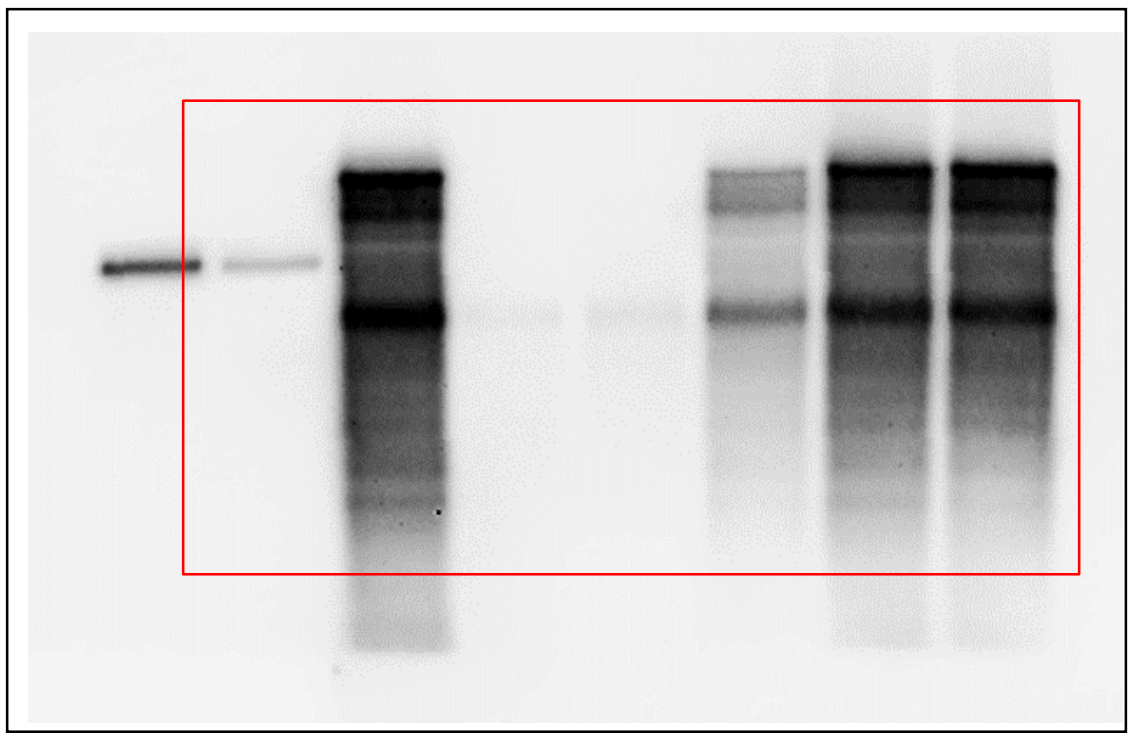

intracellular capsid-associated HBV DNA

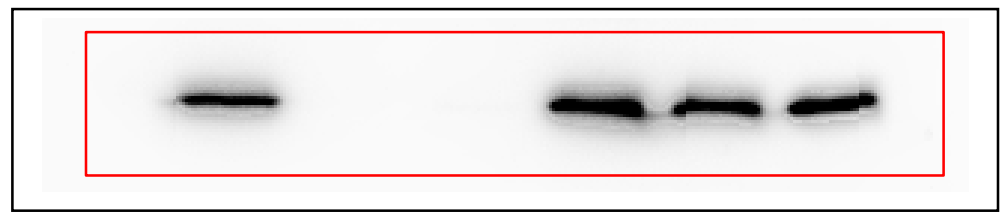

core protein

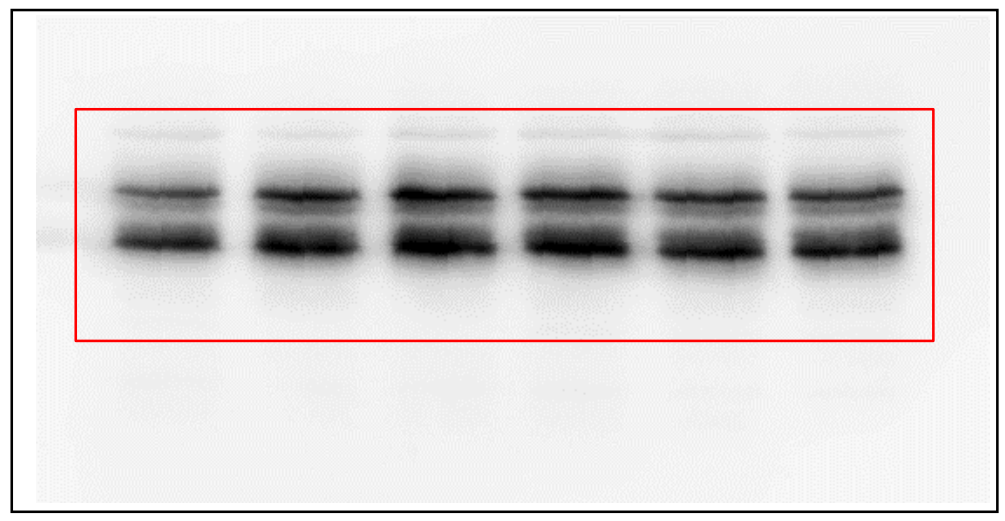

evelope proteins

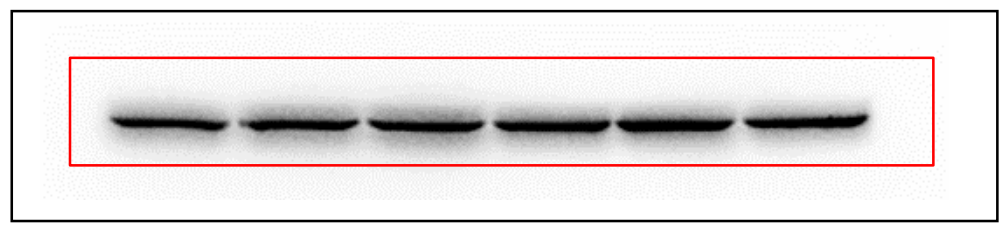

β-actin

Fig. 3C (Northern blot + agarose gel)

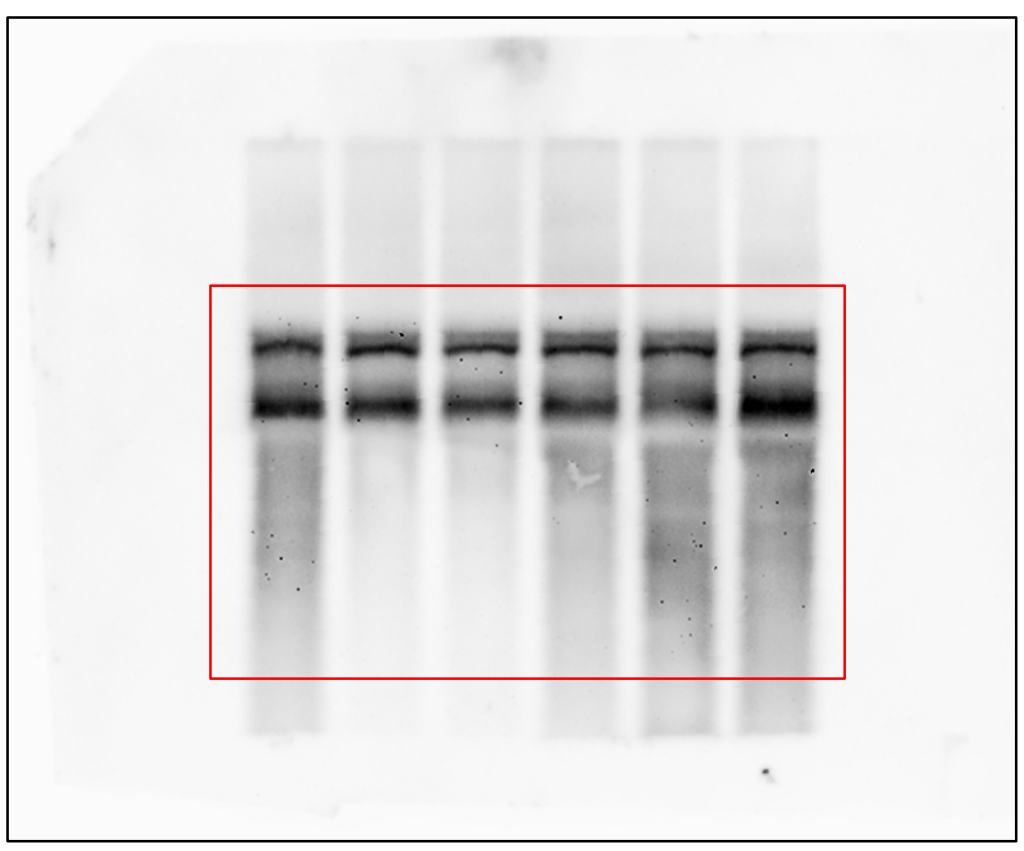

HBV RNAs (3.5 kb & 2.4/2.1 kb)

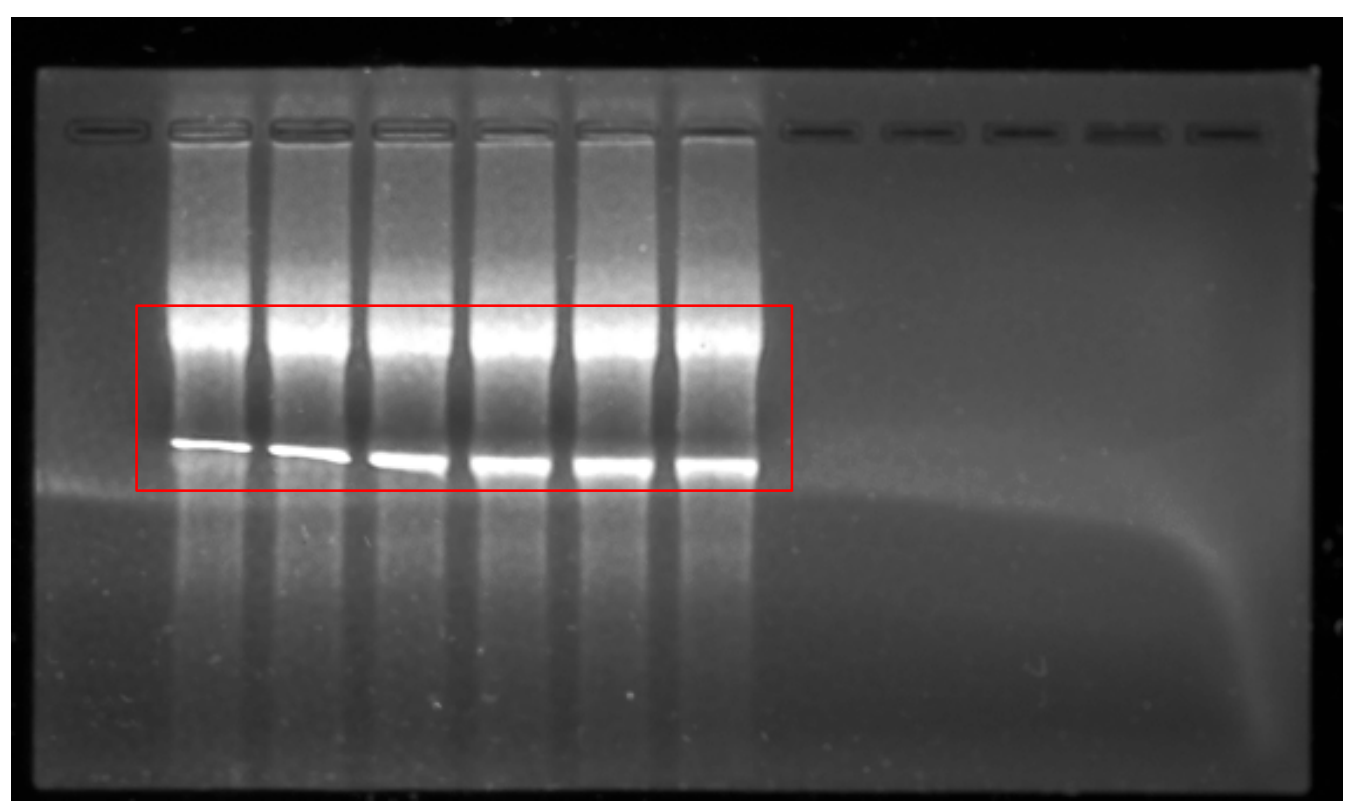

ribosomal RNAs (28S & 18S)

Fig. 3D (Southern blot)

cccDNA

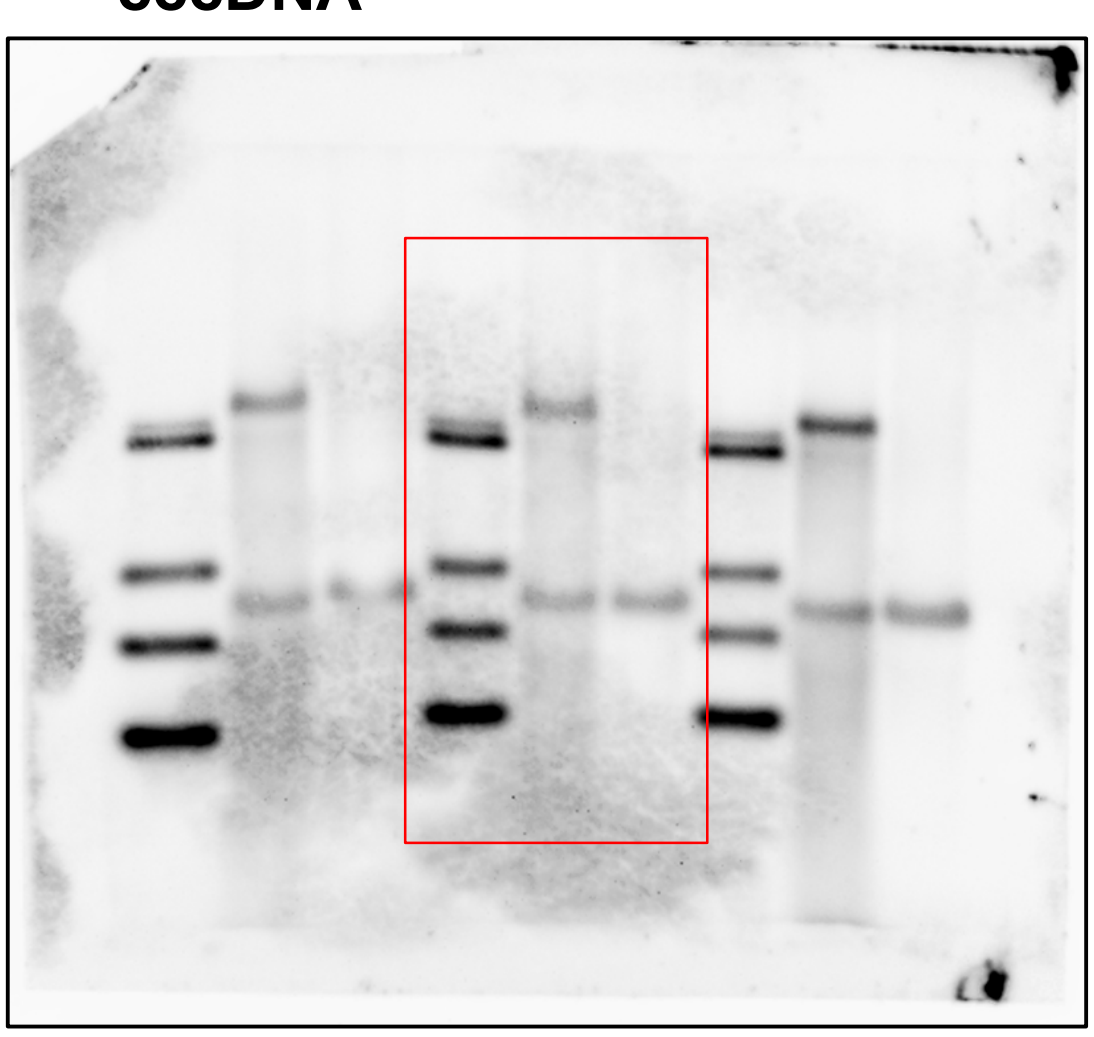

Fig. 3E (Native agarose gel)

capsid

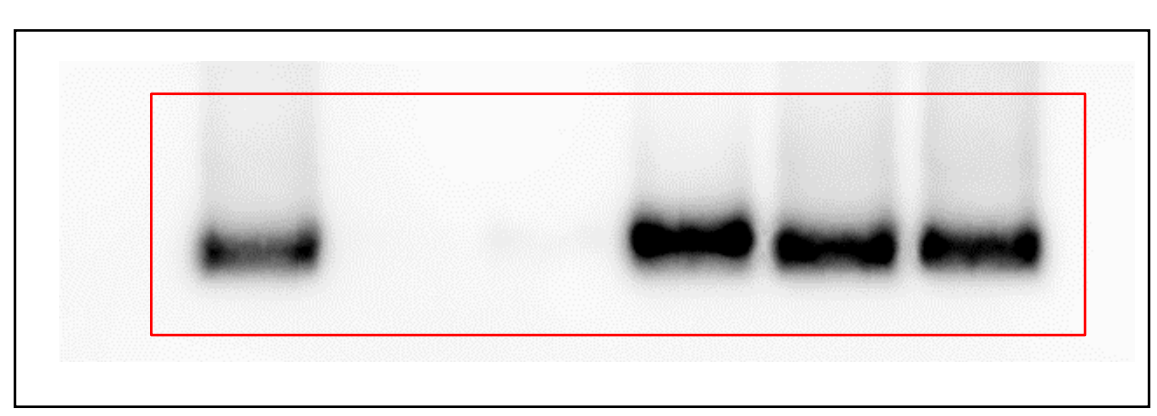

capsid DNA

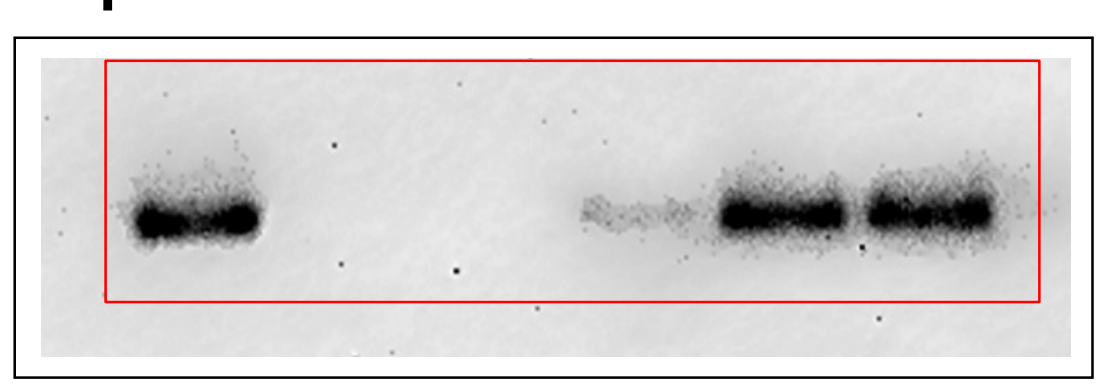

Fig. 4A (Southern blot + Westeren blot + NAGE)

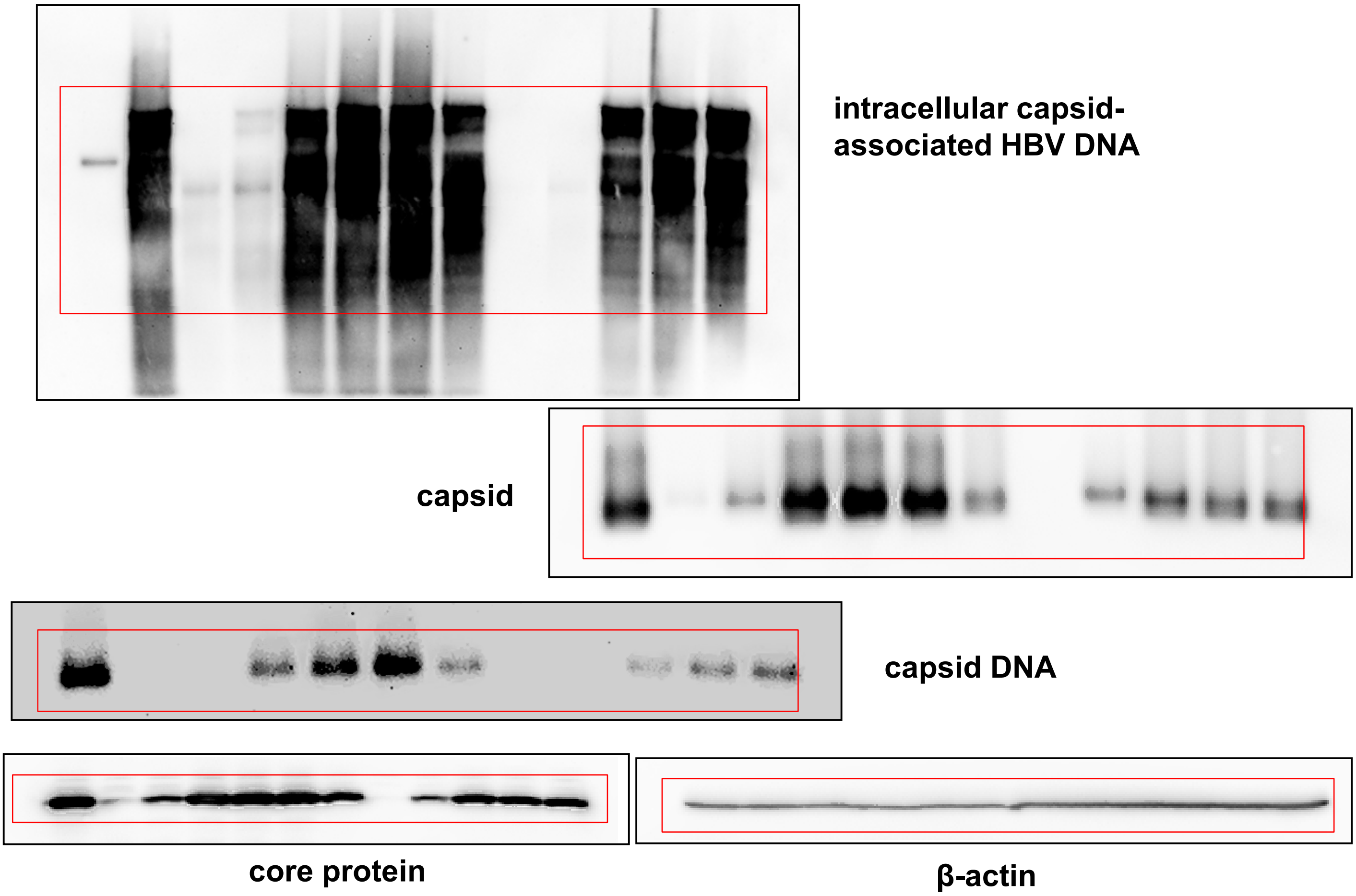

Fig. 4B (dot blot)

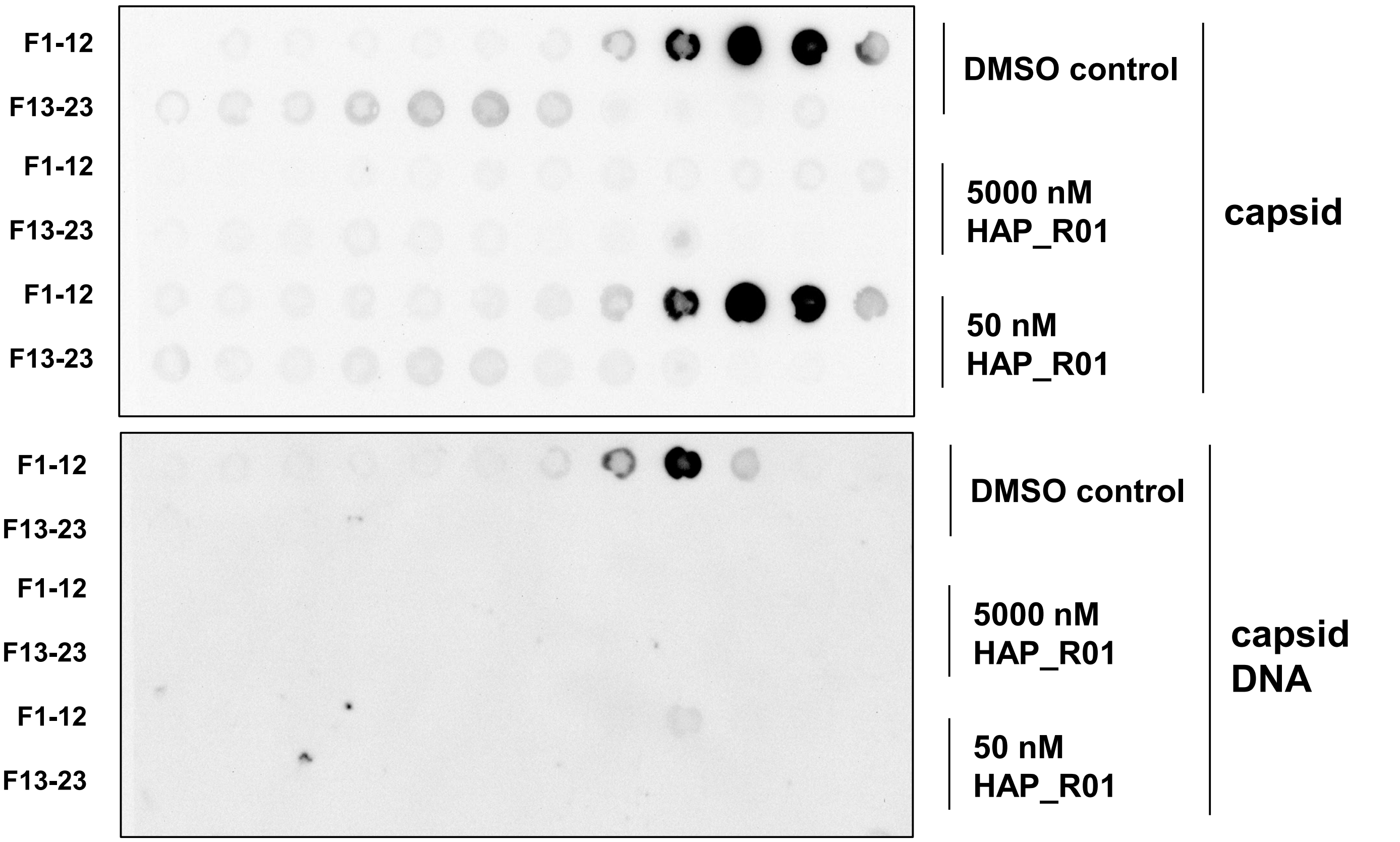

Fig. 5B-C (Southern blot + Western blot + Northern blot + NAGE)

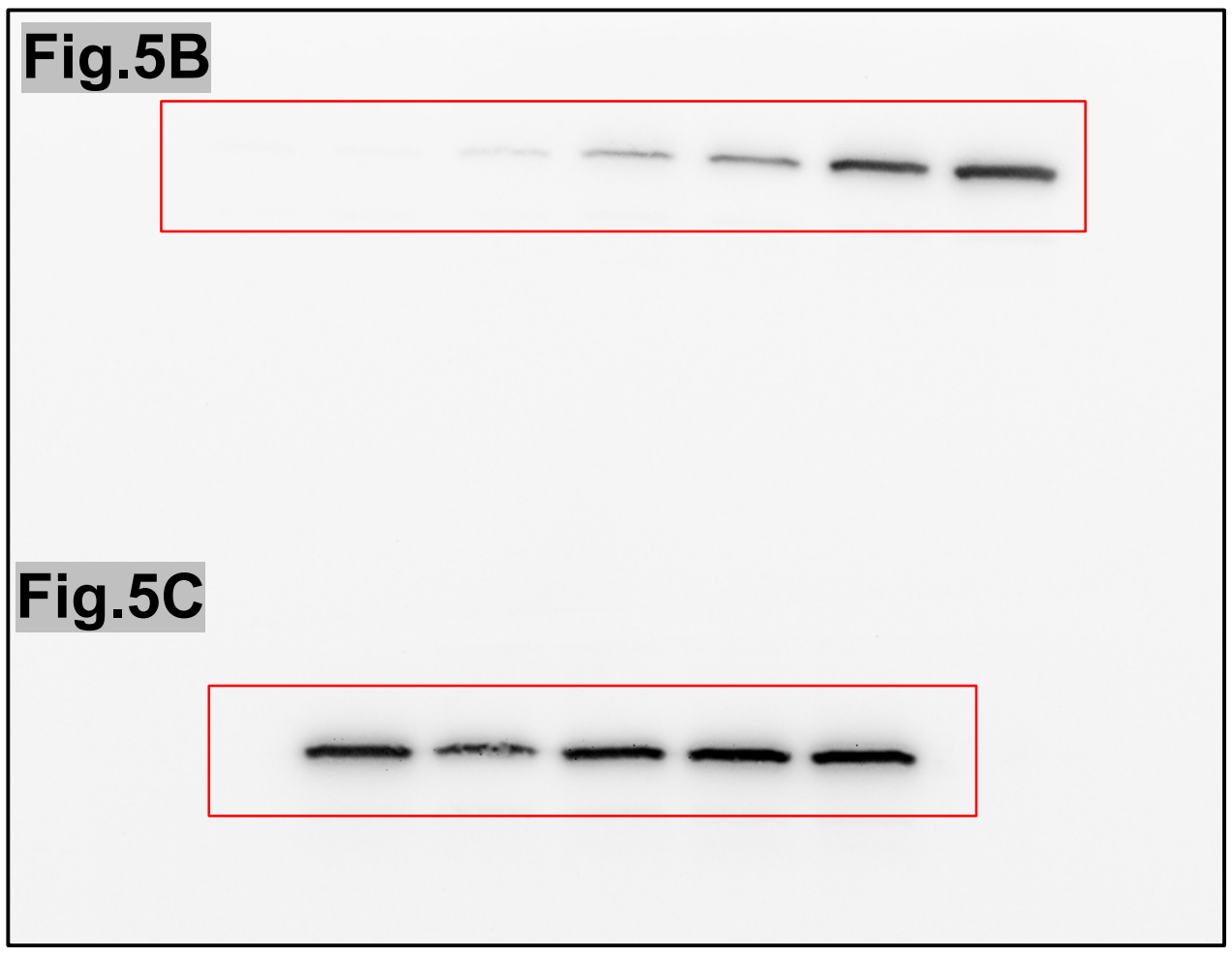

core protein

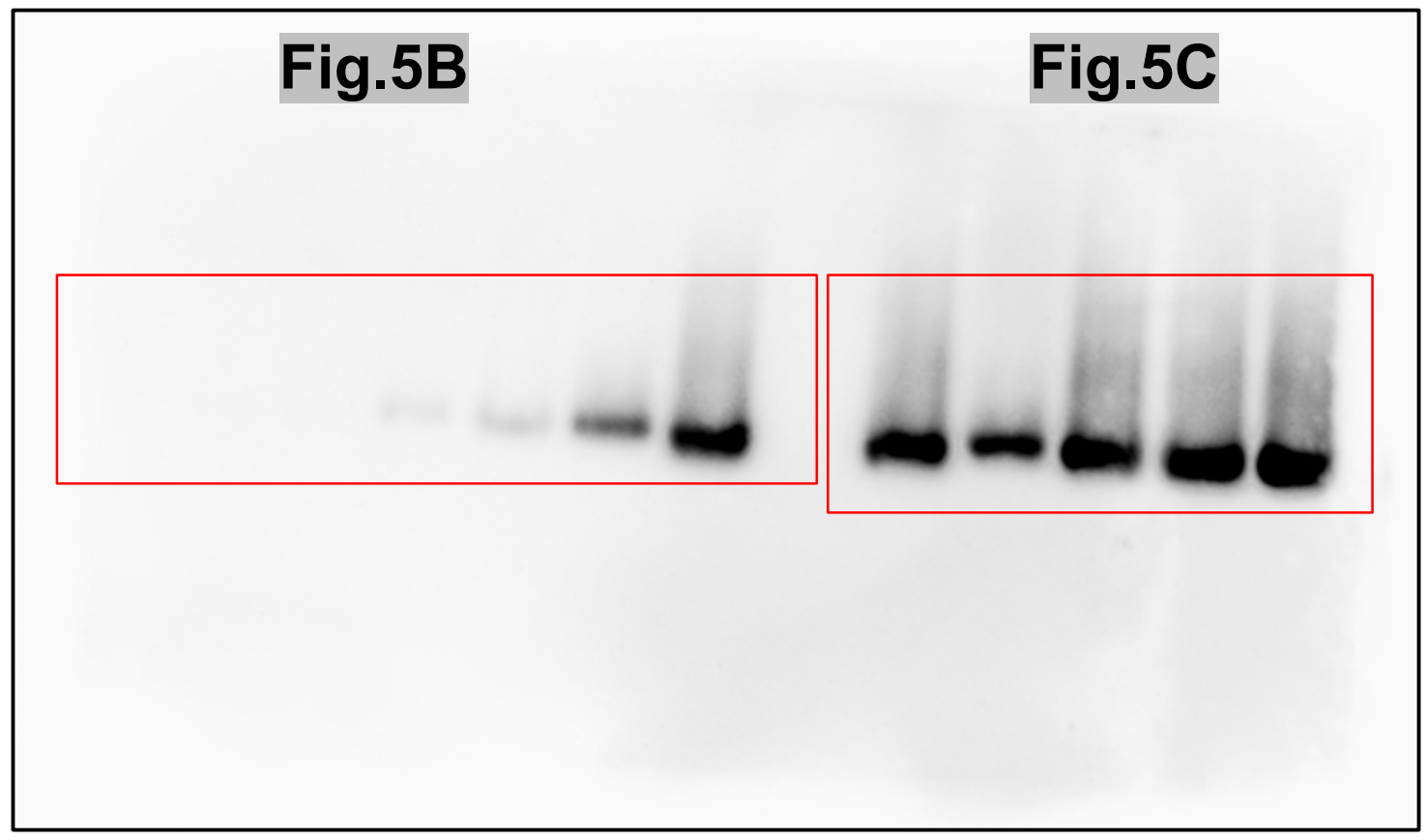

Capsid

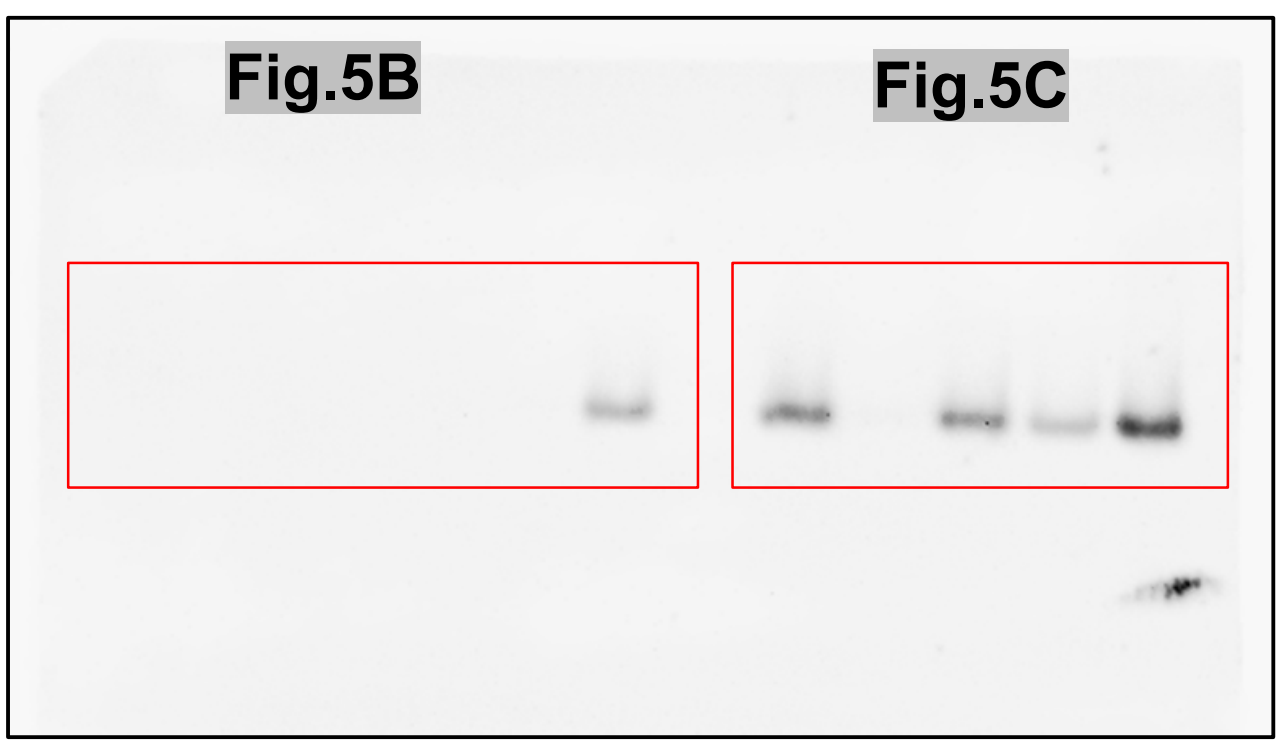

Capsid DNA

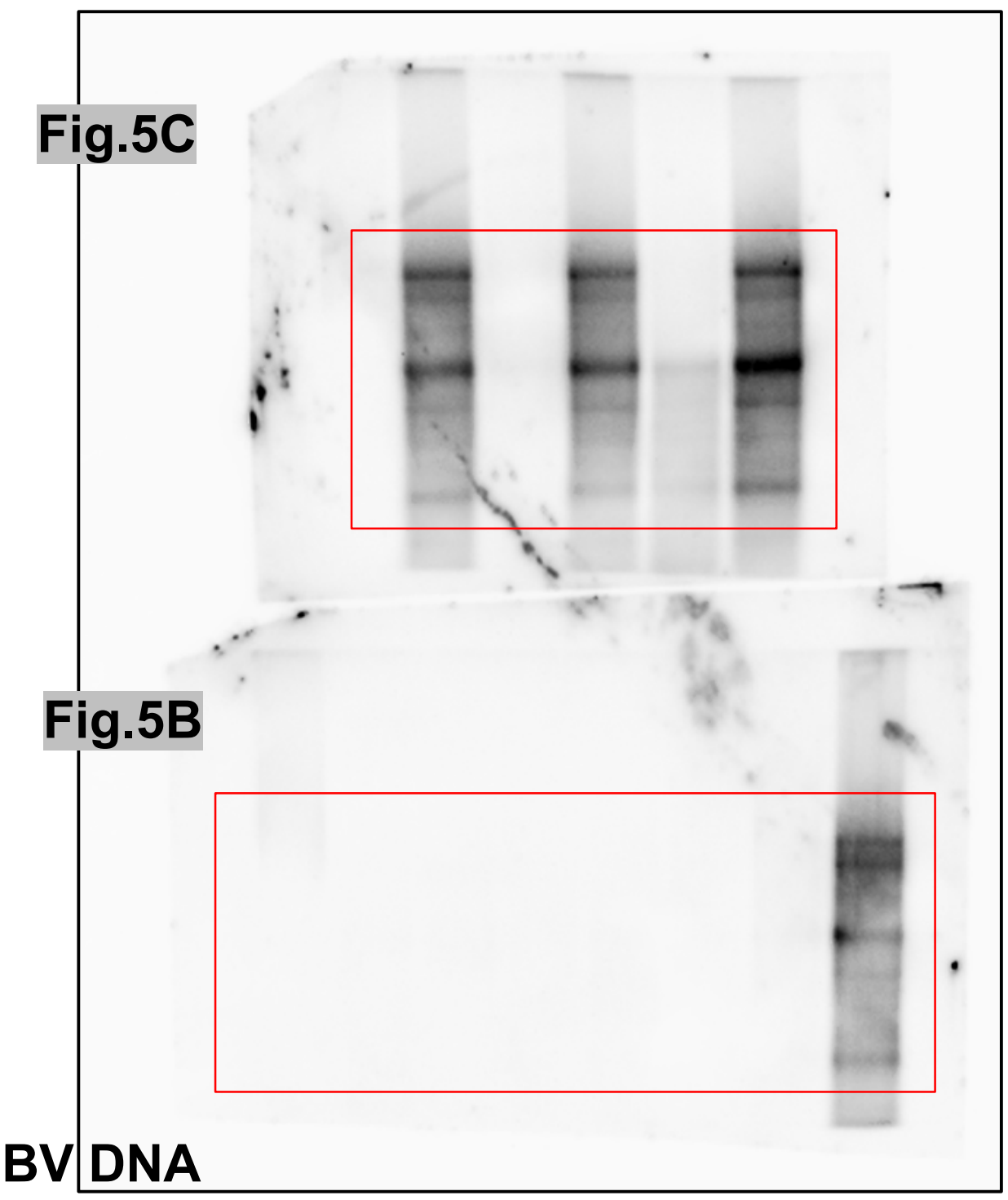

intracellular capsid-associated HBV DNA

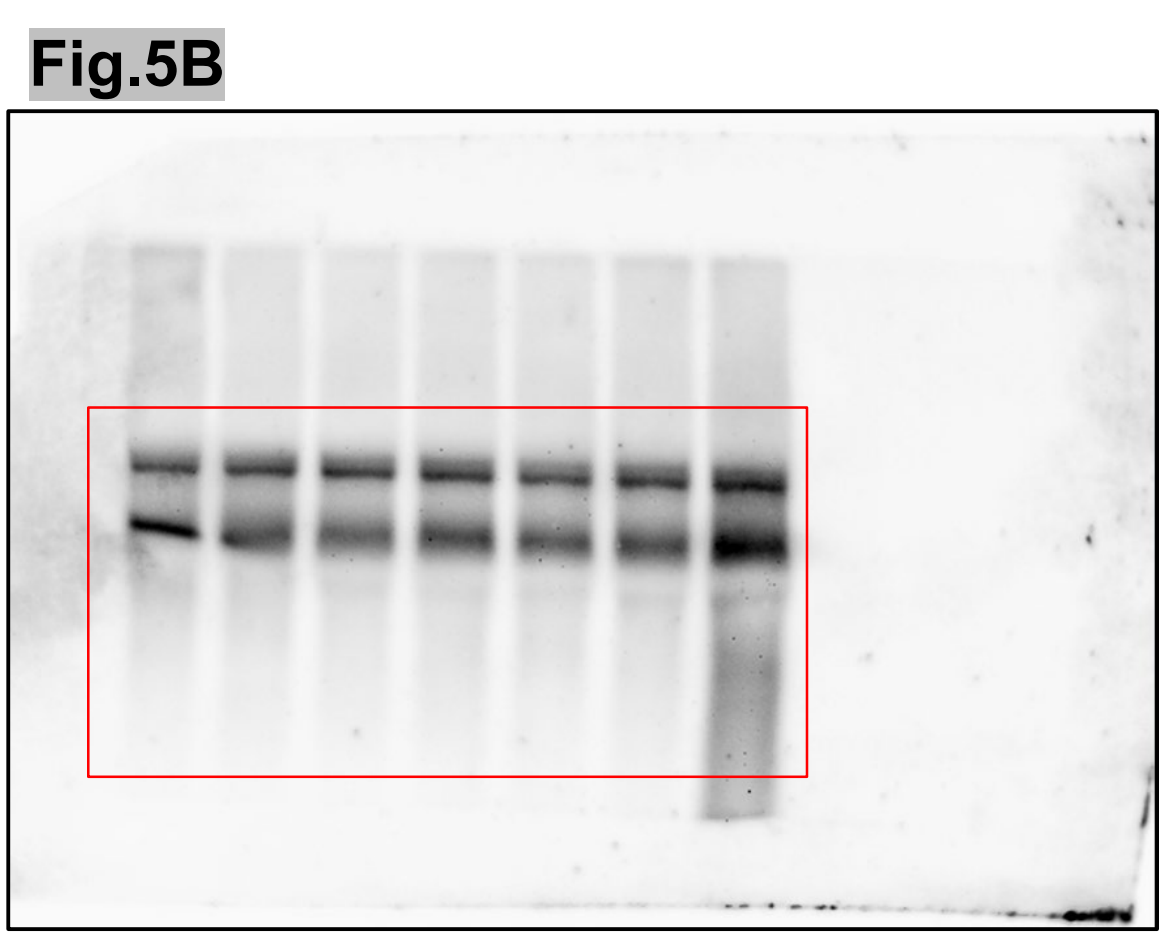

HBV RNAs (3.5 kb & 2.4/2.1 kb)

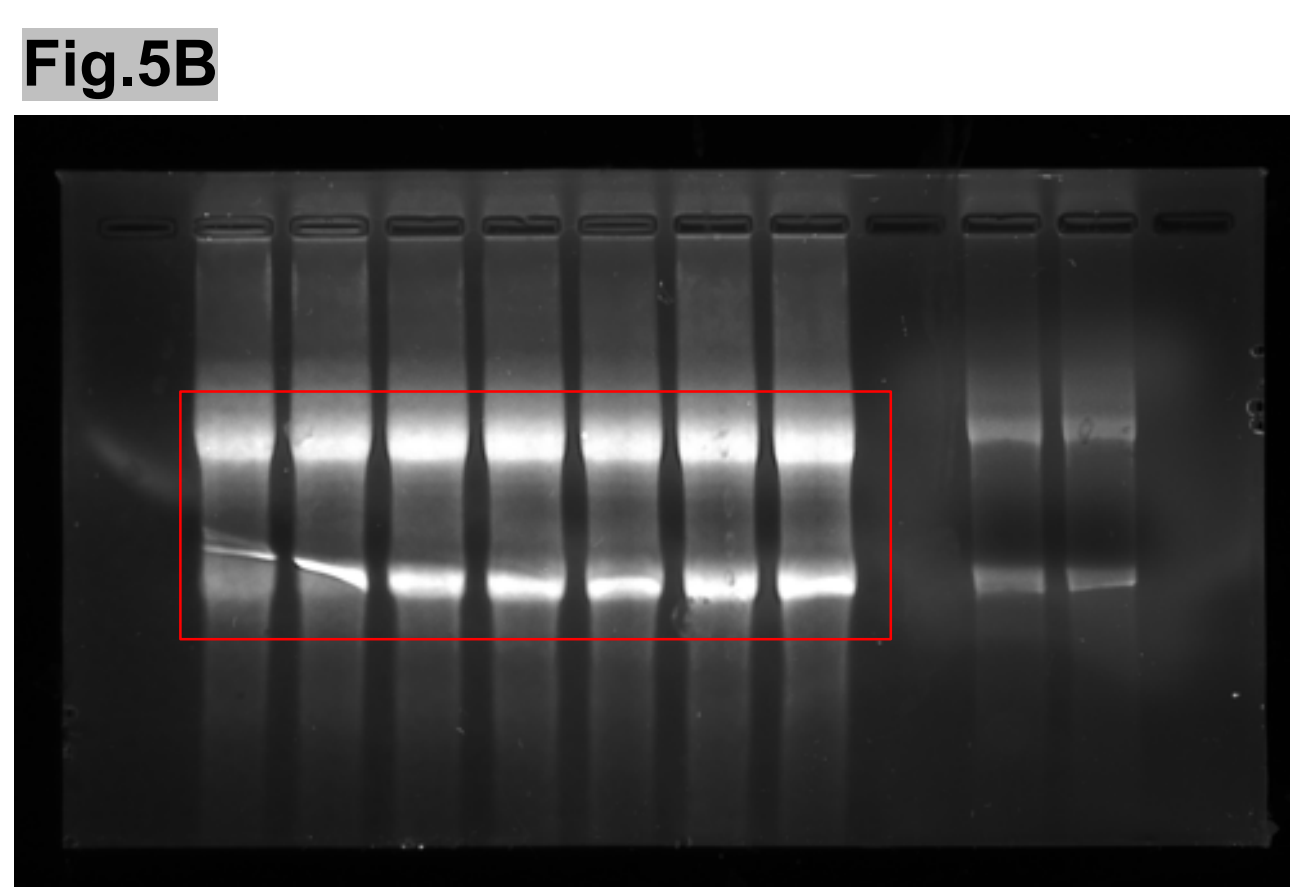

ribosomal RNAs (28S & 18S)

Fig. 7A (Western blot+native agarose gel)

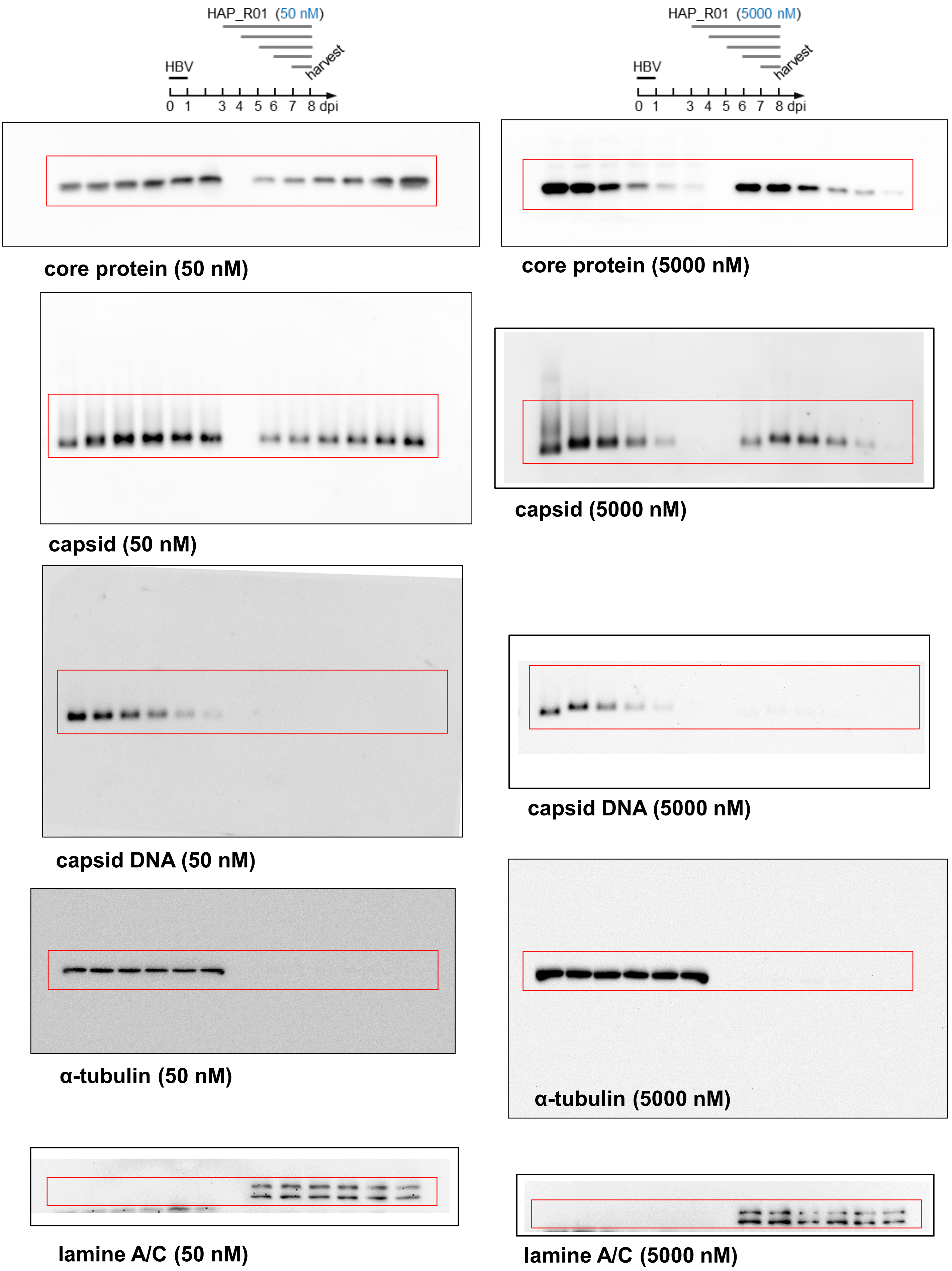

Fig. 7C (Western blot)

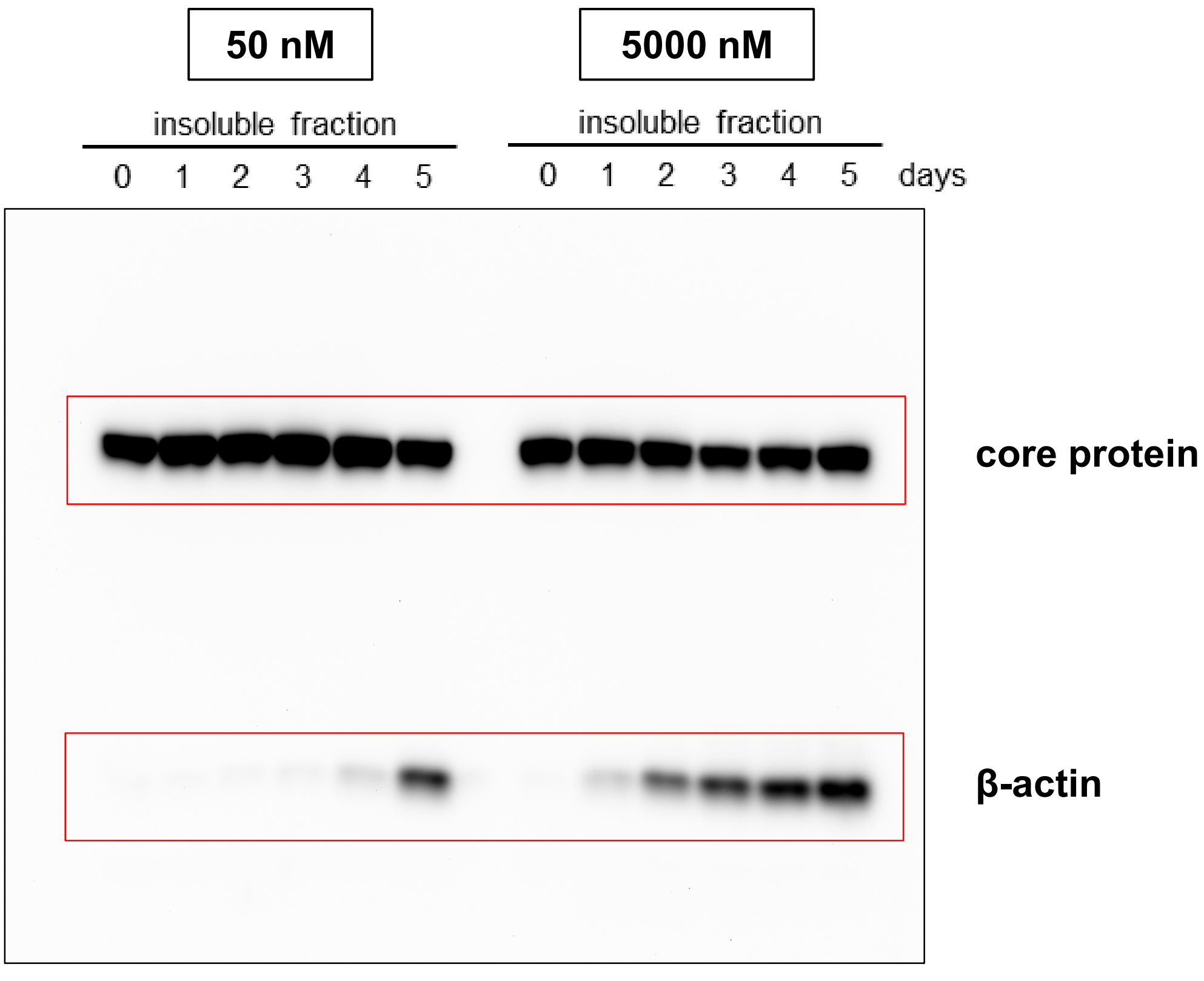

Fig. 8A (dot blot)

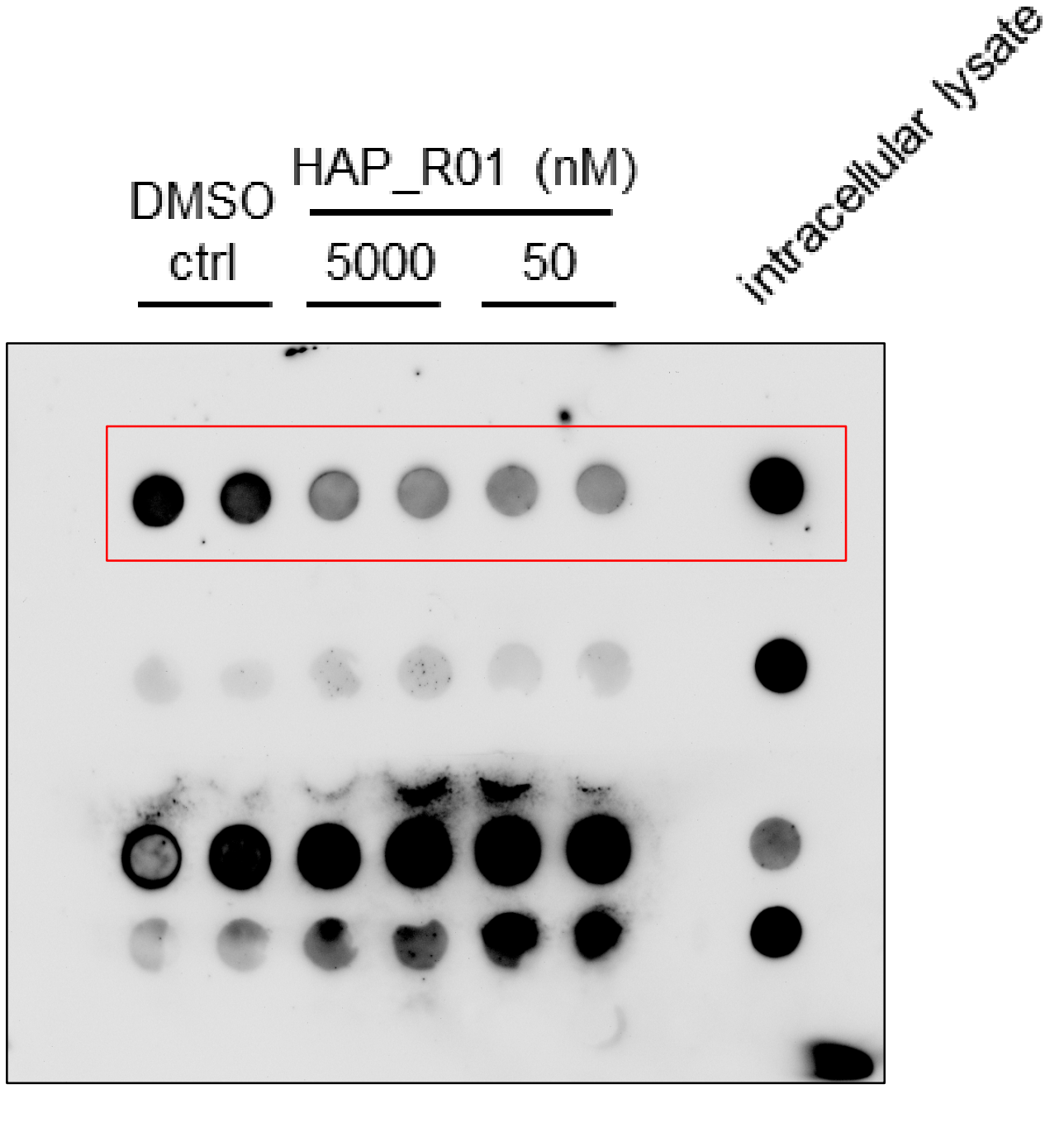

denatured extracellular HBcAg

Fig. 8B (Western blot)

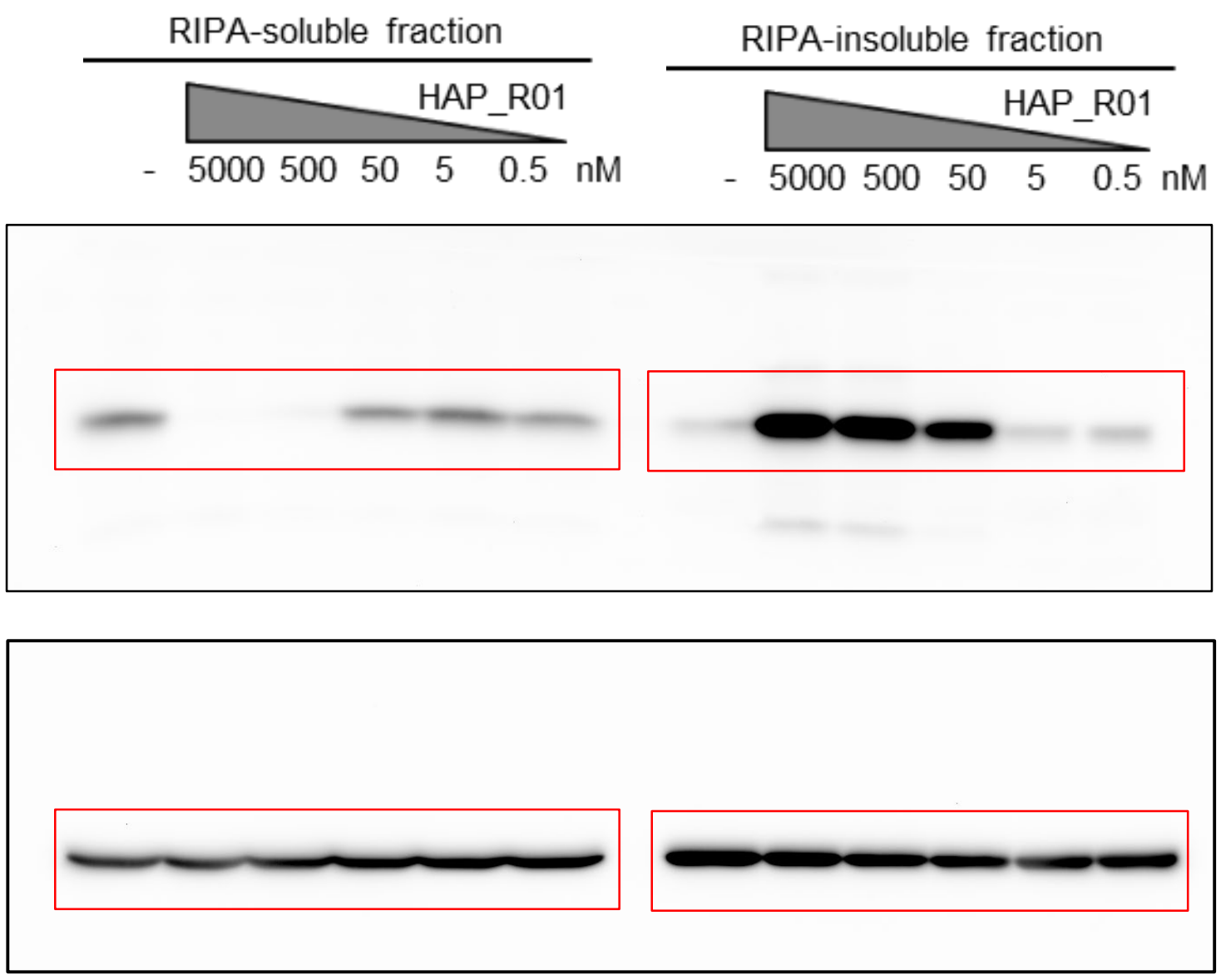

core protein

β-actin

Fig. 8C (Western blot)

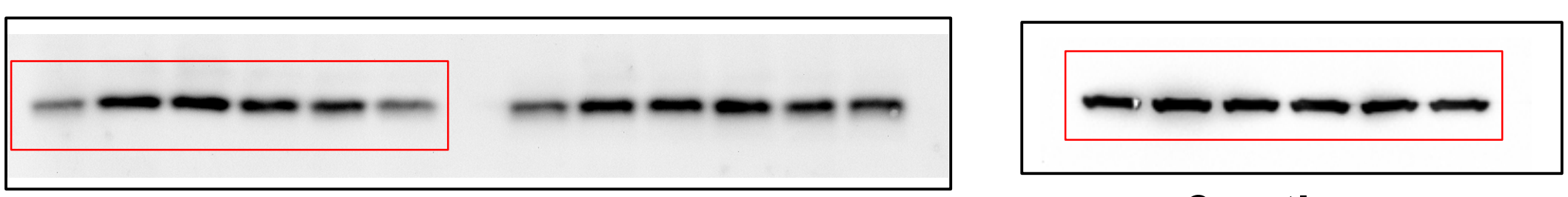

core protein

β-actin

Fig. 8D (Western blot)

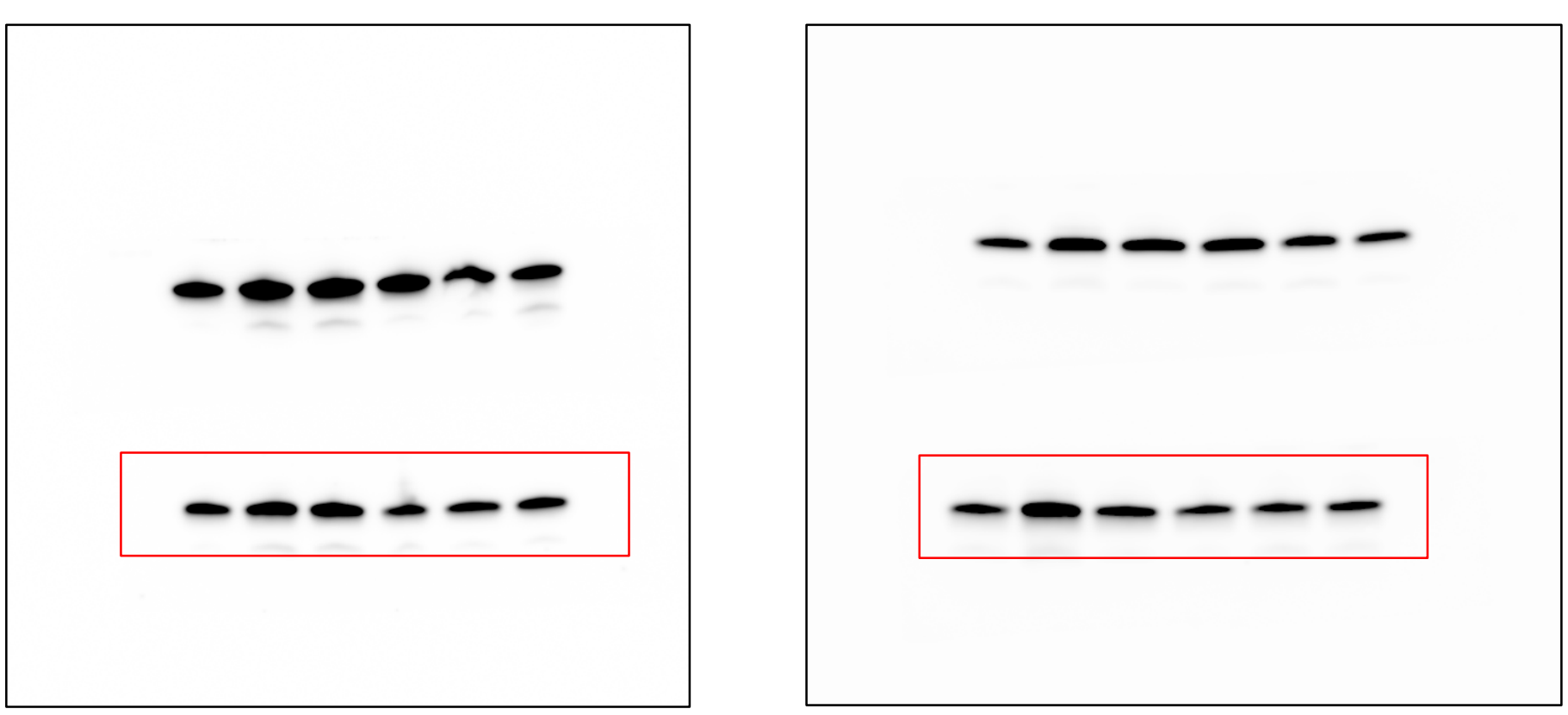

core protein

- left: Bay41-4109
- Right: GLS4

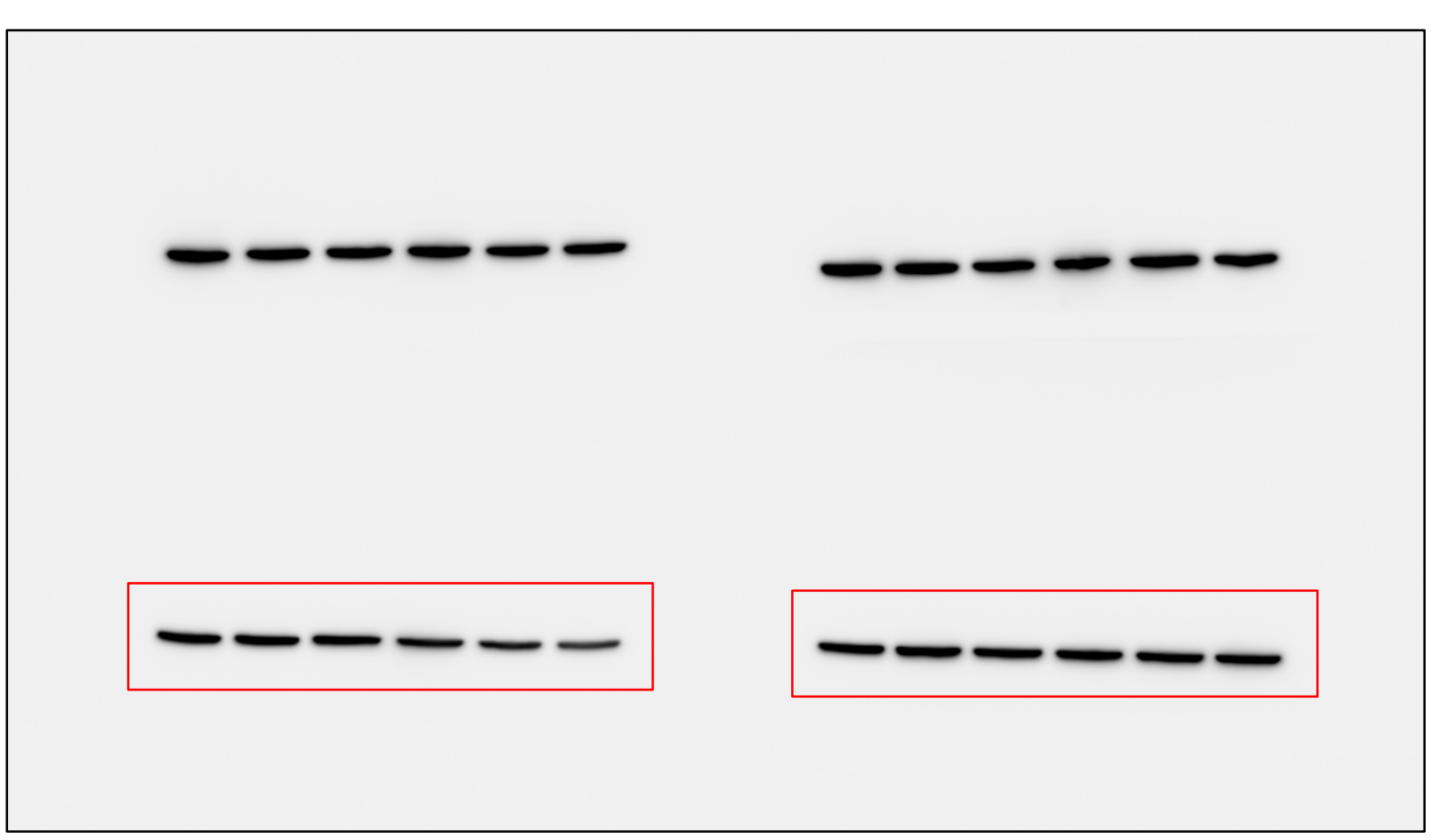

β-actin

- left: Bay41-4109
- Right: GLS4

Fig. 8E (Western blot)

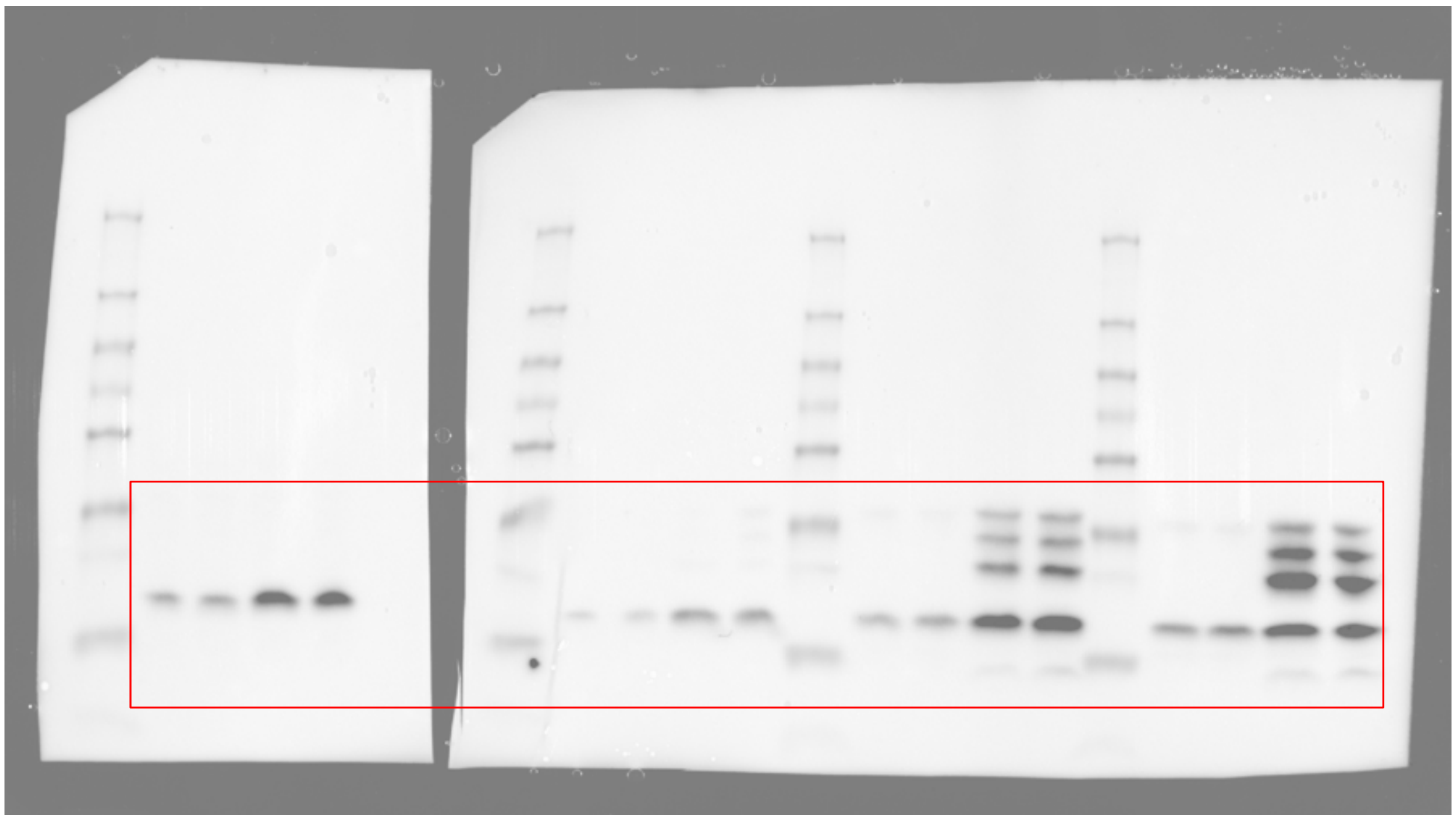

core protein

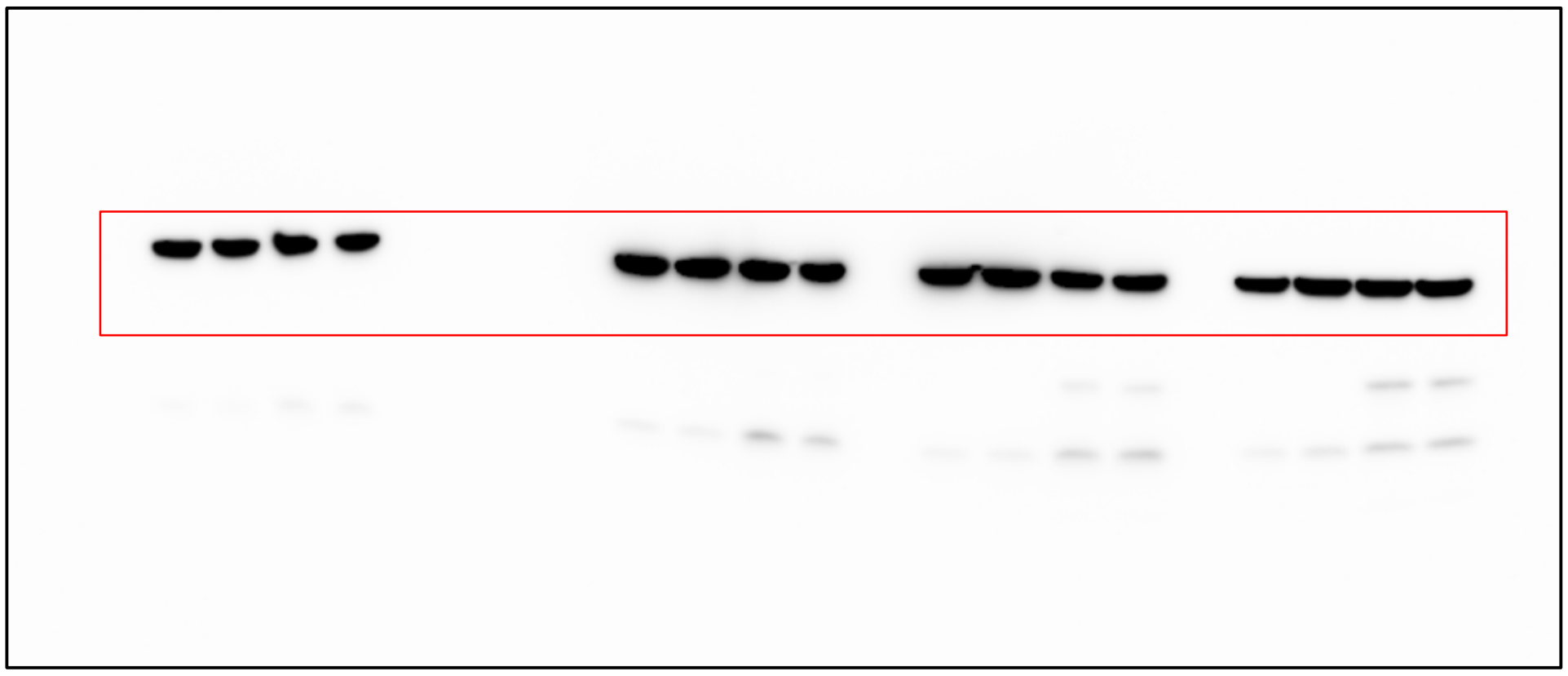

β-actin

Fig. 9C (Western blot)

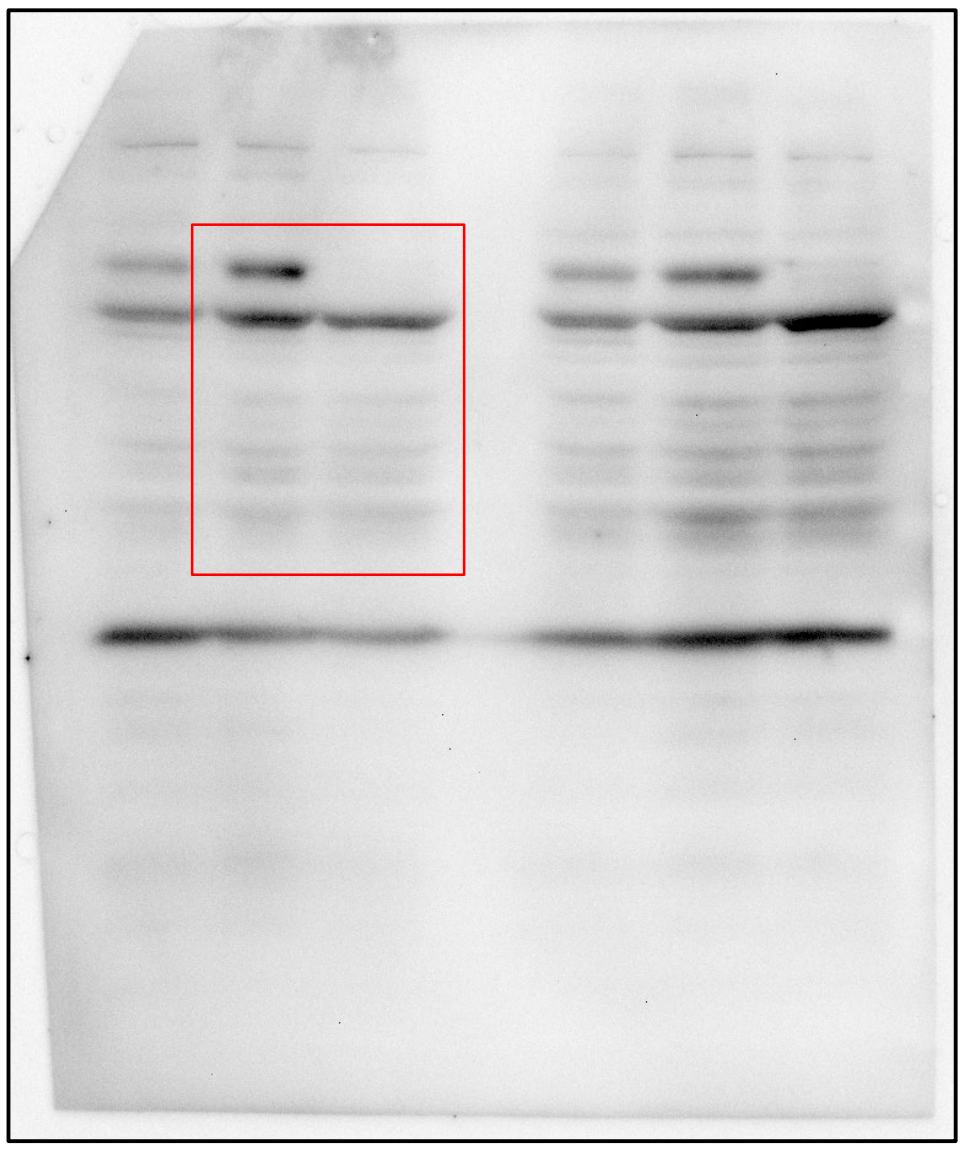

PML

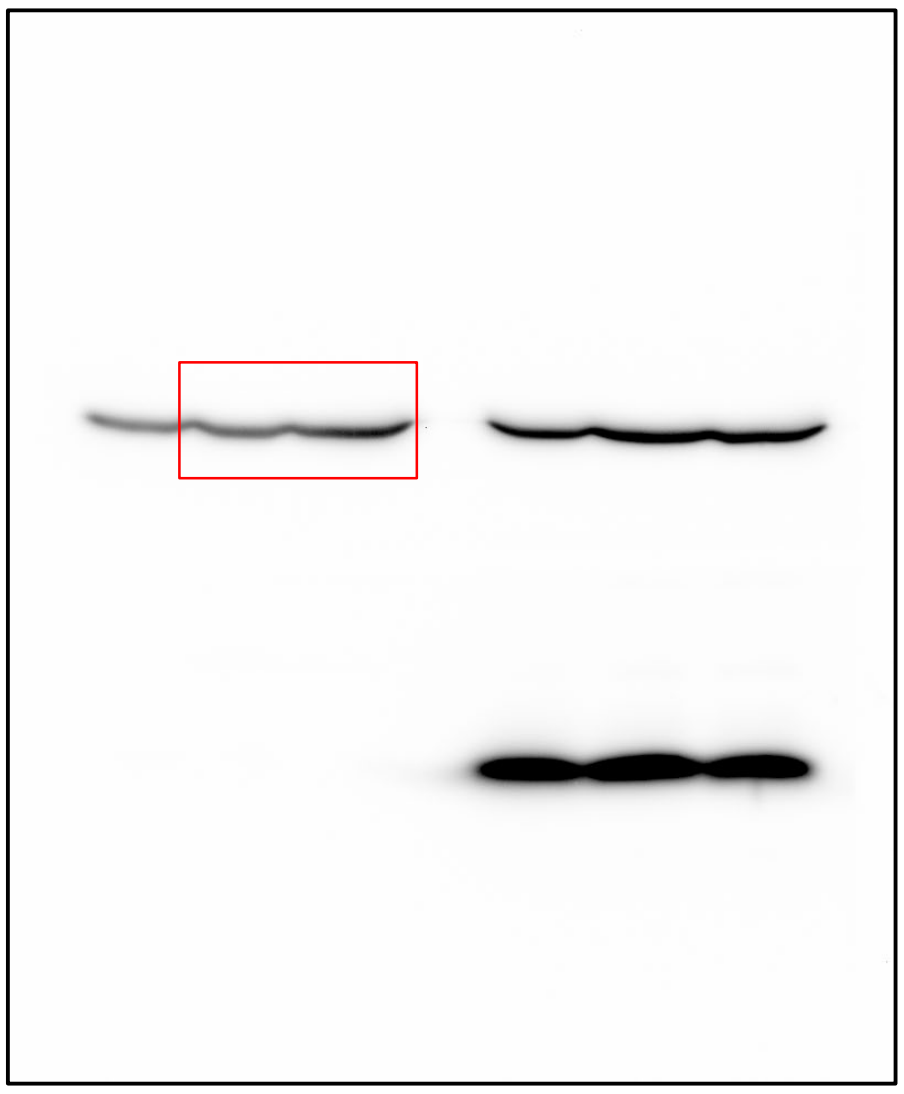

β-actin

Fig. 10A (Autoradiography)

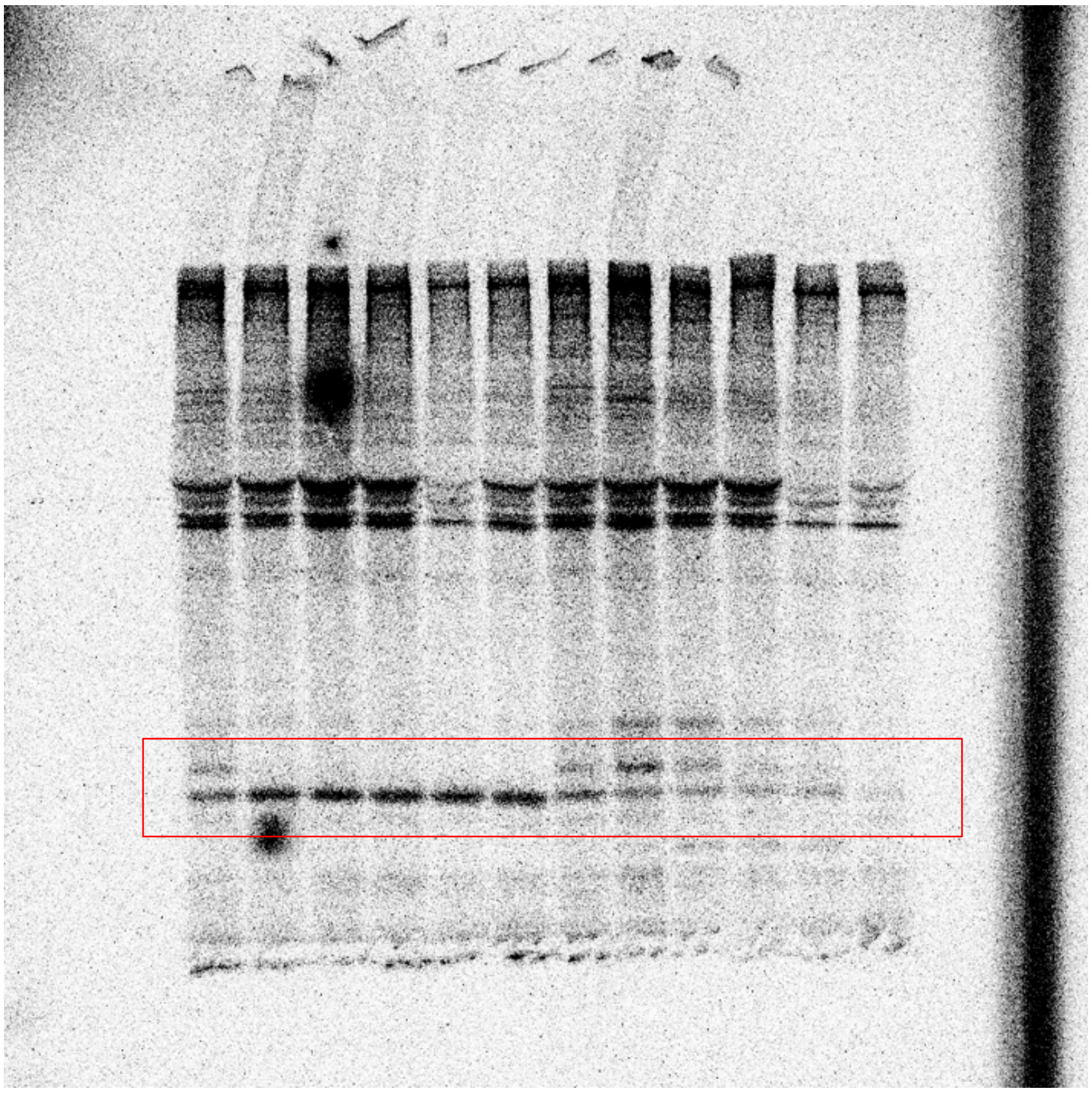

Fig. 10B (Western blot)

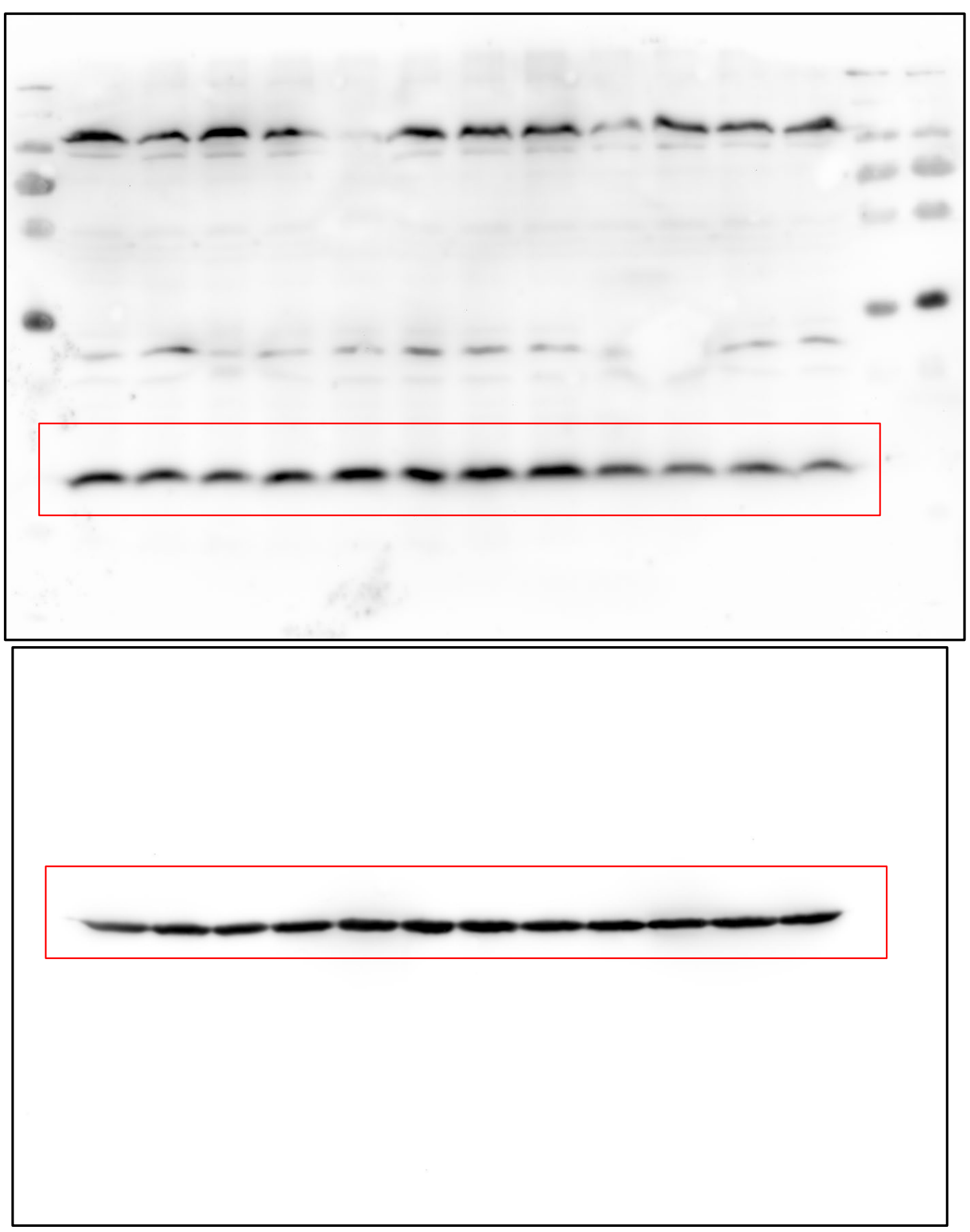

Supplement: Supplementary file 1 [file mmc1.pdf]
